# Determination of appropriate time for establishing a model of postmenopausal osteoporosis induced by bilateral oophorectomy: From bibliometric analysis to animal experiment

**DOI:** 10.1371/journal.pone.0336703

**Published:** 2025-12-04

**Authors:** Yan She, Linyu Yin, Yijin Kuang, Yujing Zhou, Dandan Zhou, Xian Tang, Shanxi Wang, Songtao Liu, Kun Ai

**Affiliations:** 1 School of Acupuncture-moxibustion, Tuina and Rehabilitation, Hunan University of Chinese Medicine, Changsha, China; 2 Affiliated Hospital of Xiangnan University, Chenzhou, Hunan, China; Australian National University, AUSTRALIA

## Abstract

**Objective:**

This study aimed to investigate the optimal age for developing a model of postmenopausal osteoporosis via bilateral oophorectomy. Moreover, the timing and effectiveness of the established model was monitored to provide scientific foundation to guide future research aimed investigating the timing and model selection in various interventions and treatments.

**Methods:**

Data from animal experiments investigating postmenopausal osteoporosis published in the past 10 years were retrieved from the Web of Science Core Collection database. The obtained data were imported into Excel, CiteSpace, and VOSviewer software and screened to identify high-frequency animal models. The sexual maturation status of these high-frequency animals at different ages was examined through vaginal smear HE staining, measurement of serum estrogen (E2) levels, and examination of ovarian uterine morphology. Bilateral ovariectomy was performed in sexually mature animals, and the HE staining assay was carried out on vaginal smears for five consecutive days starting from the fourth day post-operation to confirm the success of the ovariectomy. Subsequently, alterations in bone mineral density and bone histopathology were dynamically analyzed at 6-, 9-, and 12-weeks post-operation, which enabled us to systematically evaluate the model’s effectiveness.

**Result:**

The search identified 668 articles and bibliometric analyses demonstrated that the C57BL/6j mice were the most frequently used species, accounting for 44.3%, with the 8-week-old C57BL/6j mice being the most common (n = 139, 34.75%). Mice at different weeks of age were selected as the subjects of sexual maturation test. It was observed that there were no marked changes in estrous cycle in the vaginal smear of 4w and 6w groups, but notable cellular changes and estrous cycle fluctuations were observed in the vaginal smear of 8w, 10w and 12w. The serum level of E2 was not significantly different between the 4w and 6w groups, but the level of E2 in the 8w, 10w and 12w groups was significantly higher relative to that of the proestrus and diestrus (P < 0.05). Moreover, the ovaries of the 4w and 6w groups were smaller and flat, most of them were primordial follicles and primary follicles, without discernable a corpus luteum. The ovaries of the 8, 10 and 12w groups were enlarged and full, with mature follicles and corpus luteum detected in the 8w and 12w groups. In the time monitoring experiment, the results vaginal smear of the sham operation group revealed a complete estrus cycle, but it was irregular in the model group. Mice in the sham operation group exhibited full uterine shape, thick uterine body, which appeared fleshy pink. In addition, the uterine shape of the models in all groups following bilateral ovariectomy exhibited atrophic, irregular, thin, and gray. The trabecular bone in the model groups was disorderly, broken, and decreased compared to those of the sham operation group. At 9 and 12 weeks, the trabecular bone fracture disorder was severe in the model group; however, the number of trabecular bones was not significantly different between the 9-week model group and the 12-week model group. Furthermore, bone mineral density (BMD), bone volume fraction (BV/TV), and trabecular bone number (Tb.N) were comparable between the 6-week model group and the sham operation group. In contrast, the BMD, BV/TV, and Tb.N in the 12-week model group exhibited a modest but non-significant decrease relative to the 9-week model group. The concentration level of BALP and TRACP-5b did not differ significantly between the 6-week model group and the sham operation group. In contrast, BALP was significantly lower in the 9-week and 12-week model groups compared to levels in the sham operation group, while the levels of TRACP-5b in the 9-week and 12-week model groups were significantly higher than those in the sham operation group(P < 0.001).

**Conclusions:**

This study demonstrates that, at 8 weeks of age, C57BL/6J mice are the most suitable animal model for bilateral ovariectomy, owing to their stable maturity. A postmenopausal osteoporosis model can be established 9 weeks after the initial operation, which can facilitate further research on postmenopausal osteoporosis.

## Introduction

Postmenopausal osteoporosis (PMOP) is a common metabolic bone disease that affects postmenopausal women, manifesting as decreased bone mass and degeneration of bone microstructure [1]. The condition is an important risk factor for fractures, and its complications increase the rates of disability and mortality among patients, making it a major public health threat [2–4]. To determine the pathophysiological mechanisms associated with PMOP and develop potential therapeutic strategies, it is imperative to establish a reliable animal model [5].

The ovariectomy (OVX) model has been shown to accurately replicate the features of estrogen-deficient state seen in postmenopausal women [6,7], and the associated bone responses are analogous to those detected in this population, including the occurrence of bone loss [8]. Therefore, it is used as a standard experimental model for investigating postmenopausal osteoporosis (PMOP) [9]. Studies have shown that the effects of estrogen replacement therapy differ significantly between the early and late stages following OVX surgery [10,11], suggesting that the timing of estrogen deficiency may have profound effects on the phenotypes of the PMOP model. Moreover, significant variability exists in species, ages, and postoperative observation periods regarding the design of the PMOP models under various studies [12–15]. This inconsistency in modeling standards can reduce the comparability of experimental outcomes and their clinical applicability, thereby weaking the reliability of the models in disease mechanism exploration and treatment evaluation.

In recent years, bibliometrics, which is a quantitative research method based on the analysis of large-scale literature data, has revolutionized the research in various fields, allowing the identification of core scientific issues, and determination of the prevalence of research practices [16–18]. In previous studies, model parameters for PMOP are often selected based on the experience of individual laboratories, the historical legacies of specific studies, or the interpretation of few representative publications, implying the lack of systematic and objective criteria. For this reason, there is a high degree of heterogeneity in model selection, which severely affecting the comparability and reproducibility of experimental results [19]. Using the available databases and other related data, bibliometrics can reveal the mainstream approaches employed in diverse research communities, thereby providing a consensus-based, evidence-driven reference for model standardization [20]. Compared to traditional empirical selection or small-scale literature reviews, this method is systematic, objective, and data-driven.

Therefore, we screened the various species and ages of commonly used animals leveraging bibliometric methods and to identify the optimal surgical window for model animal. Furthermore, the timing of significant bone loss in the OVX-induced PMOP model was investigated by dynamically monitoring of postoperative uterine morphology, micro-computed tomography (micro-CT), and bone histopathological changes, thereby uncovering important scientific insights that will optimize model construction standards and identify the key time points for treatment. The outcomes of this study are expected to enhance the standardization of PMOP animal models and lay a crucial foundation for subsequent mechanistic investigations and drug development.

## Bibliometric study on postmenopausal osteoporosis in the past decade

To reveal the prevalence trend of the use of animal models for postmenopausal osteoporosis over the past decade and identify the most preferred animal species in this field, we adopted a bibliometric approach to evaluate relevant literature published between 2015 and 2025.

### Materials and methods

#### Literature materials.

**Fig 1:**
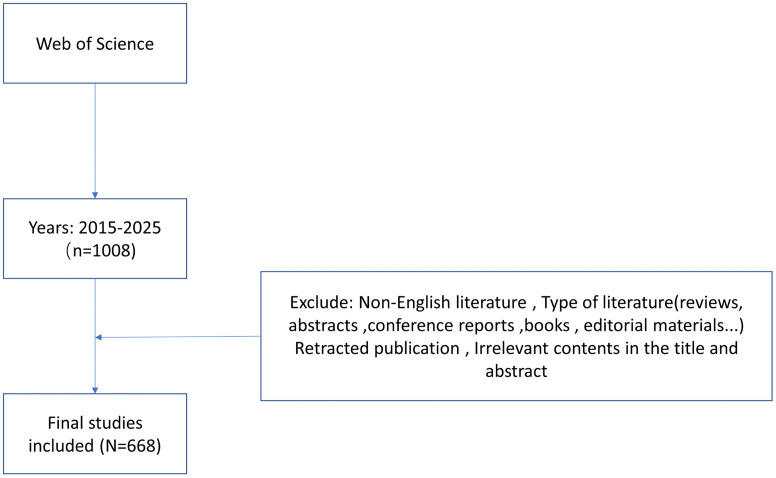
Flow chart of data screening and analysis.

The Web of Science Core Collection database was searched to identify relevant articles from 2015 to 2025 focusing on animal experiments targeting postmenopausal osteoporosis. The retrieved articles were screened and any differences between the two authors were resolved through consensus. The data were imported into Excel, Citespace, and VOSviewer software for subsequent analyses in terms of the type of animal model, country/region, institution, journal, author, keywords, and references. The obtained data were used to create a knowledge map. The identified articles were searched using the established subject headings “postmenopausal osteoporosis” and “animal models.” Next, full texts were reviewed, and articles that met the pre-determined criteria were stored in plain text format together with their complete records and citations. The materials were imported into the Citespace and VOSviewer for data conversion and quantitative analysis, and the literature was deduplicated using the software’s deduplication function. The screening identified 668 articles, from which data regarding animal models were manually extracted and entered into Microsoft Office Excel 2019 to establish a database for further induction and analysis(Fig 1).

#### Statistical methods.

Microsoft Office Excel 2019, CiteSpace, and VOSviewer were used to visualize the literature data retrieved from the Web of Science Core Collection database, while all statistical analyses were performed using GraphPad Prism 10 software.

### Result

**Fig 2:**
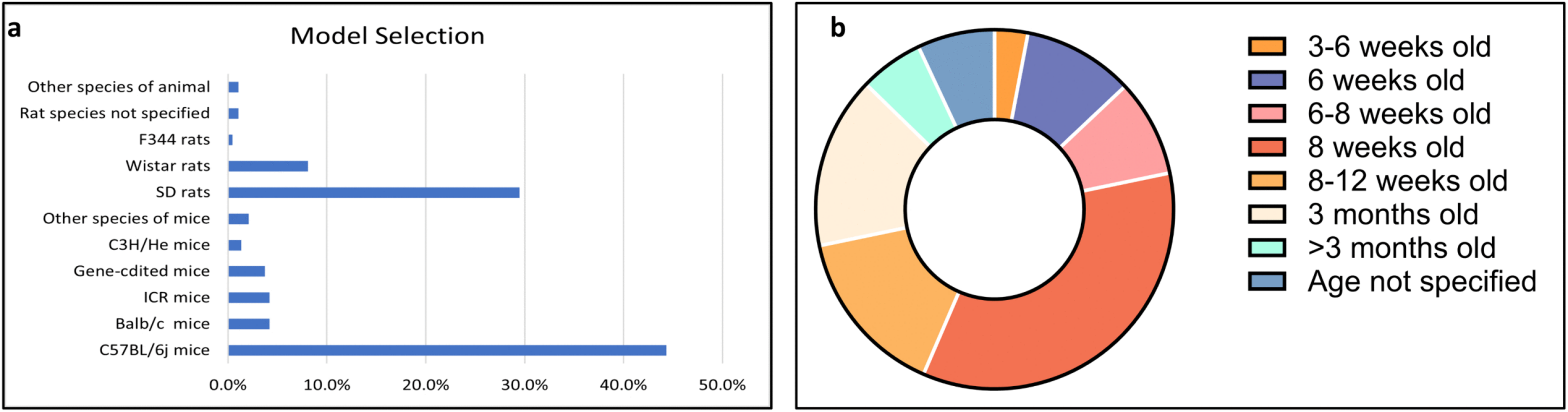
Presents the bibliometric search results. (a) illustrates the proportion of model species selected, (b) depicts the age classification of C57BL/6j mice utilized for constructing PMOP models through ovariectomy (OVX).

In the current bibliometric analysis, 668 articles related to animal models of postmenopausal osteoporosis and the species-specific analysis of the models were included. There were specific-specific differences among the selected model species. Moreover, the use of C57BL/6j mice was significantly higher than that of other species, accounting for 44.3% of the total. This was followed by SD rats at 29.5%, and the remaining species accounted for relatively smaller proportions (Fig 2a). Analysis of age distribution of C57BL/6j mice. demonstrated that the most frequently used age group for modeling was 8 weeks (n = 139, 34.75%), followed by the 8–12 weeks and 3 months age groups, each comprising around 15% of the total (Fig 2b).

## Experiment on the sexual maturation time of C57BL/6J mice

Based on the conclusions of the aforementioned bibliometric studies on postmenopausal osteoporosis over the past decade, C57BL/6J mice are the most widely used strain in PMOP research. We selected C57BL/6J mice as the research subjects to explore their sexual maturation time, thereby providing a basis for determining the optimal timing of ovariectomy in model mice.

### Materials and methods

#### Experimental animals.

Five female C57BL/6J mice, aged 4 weeks (4w), 6 weeks (6w), 8 weeks (8w), 10 weeks (10w), and 12 weeks (12w), were purchased from Hunan Slake Jingda Laboratory Animal Co., Ltd. (license number: SYXK (Xiang) 2019–0009). The mice were housed in cages, with four mice per cage, grouped by age, and allowed unlimited access to water and a standard diet. The room temperature was maintained at 24–26 °C, the humidity was set at 55%−60%, and lights were provided for an appropriate duration. The mice cages were kept in a specific pathogen-free (SPF) laboratory environment. Each mouse received an equal quantity of the basic diet and was provided with adequate drinking water. The experiment was approved by the Animal Ethics Committee of Hunan University of Traditional Chinese Medicine, and all animal care and experimental procedures were consistent with the Guiding Opinions on the Good Treatment of Laboratory Animals issued by the Ministry of Science and Technology of the People’s Republic of China.

#### Main reagents and instruments.

The following materials were utilized in the experiments: physiological saline, hematoxylin and eosin (HE) dye, a manual single-channel adjustable pipette (Dragon, China) with a volume range of 0.5–10 µL, adhesion slides (Citotest, China, Item No. 188105), micro hematocrit capillary glass blood collection tubes (Kimble, America), an estradiol ELISA kit (Wuhan USCN Business Co., Ltd., China, Item No.: USCN-CEA461Ge), and anticoagulant centrifuge tubes(Jiannai, China).

#### Grouping of animals.

The mice were divided into groups based on their age as follows: 4-week, 6-week, 8-week, 10-week, and 12-week groups.The general health status of the mice was assessed by examining coat condition, feeding behavior, and the presence of visible trauma or deformities, in addition to measuring body weight. Mice exhibiting a body weight more than 20% below the mean value of their respective cohort were classified as abnormal. According to this criterion, one mouse from the 4-week group and one from the 6-week group were excluded from further analysis. The remaining mice were subjected to vaginal exfoliative cytology smear tests and blood analysis. During the blood collection procedure, one mouse died in the 8-week and 10-week groups.

#### Indicators of observation.

(1) Vaginal exfoliated cell smear

Four mice were selected from different age groups each day at 9 am. Next, 10 µL of normal saline was drawn using a 0.5–10 µL pipette and administered into the vagina five to six times. The induced vaginal secretions were collected and placed on an adhesive slide, and a cover glass was used for scraping to ensure a uniform spread of the secretion [21]. The slides were allowed to air dry before staining with HE once daily for 14 consecutive days.

(2) Dynamic detection of serum estradiol concentration

To determine the concentration of estradiol, a vaginal smear was performed and the mice were placed in an induction chamber pre-filled with 5% isoflurane vapor for anesthetization at a flow rate of 1 L/min. Once the righting reflex was lost, the anesthesia was maintained using a nose cone delivering 2% isoflurane in oxygen at a flow rate of 0.5 L/min. Under this anesthesia, 300 µL of orbital blood was collected daily for six consecutive days. The concentration of E2 as determined and plotted against the vaginal smear results to determine the changes in E2 levels throughout the estrous cycle. The collected serum was centrifuged at 1,500 rpm with a radius of 7 cm for 20 minutes at 4 °C, and the supernatant was carefully collected and subjected to the Enzyme-linked immunosorbent assay (ELISA) to quantify the dynamic changes in E2 levels during the estrous cycle.

(3) Ovarian histological examination

The mice were euthanized via cervical dislocation and three ovarian samples were randomly collected from each group for histological assessment. The samples were fixed in 4% neutral formaldehyde, embedded in paraffin, and sectioned, followed by HE staining in line with the manufacturer’s instructions.

#### Statistical methods.

For the analysis of dynamic estrogen level data, a two-way repeated-measures ANOVA was employed. Continuous data are expressed as mean ± standard deviation (mean ± SD). The Geisser–Greenhouse correction was applied to adjust for violations of sphericity. In cases where a significant main effect or interaction was identified (P < 0.05), Tukey’s post hoc test was used for pairwise comparisons between groups. All statistical analyses were conducted using GraphPad Prism software (version 10), with a P-value less than 0.05 considered statistically significant.

### Result

**Fig 3:**
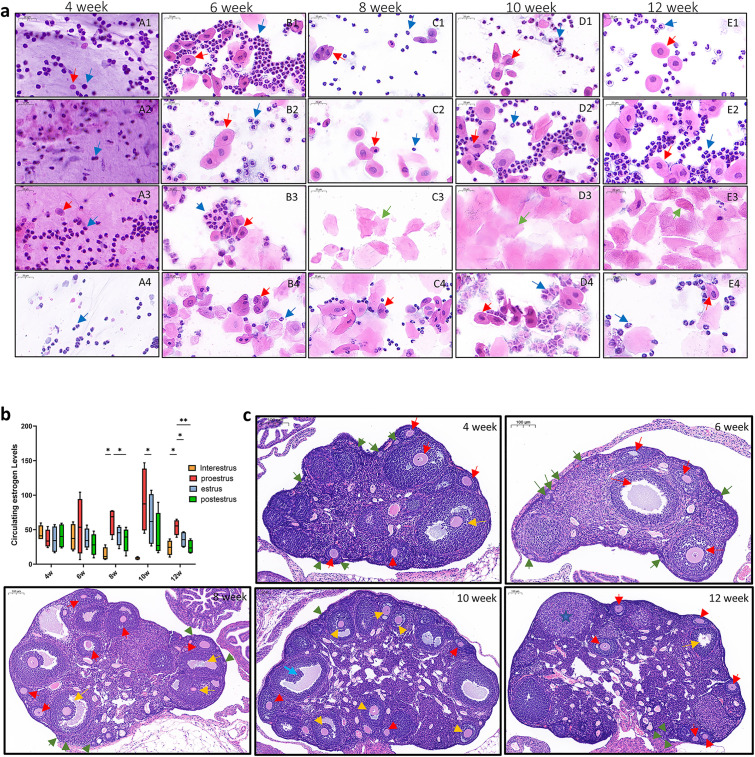
Sexual maturity status of female C57BL/6J Mice at different ages. **(a)** The variations in vaginal exfoliated cells across different weeks of age are presented (50 × , n = 3). A1-A4 corresponds to the estrus interval in the 4-week-old group; B1-B4 represents proestrus in the 6-week-old group; C1-C4 delineates the phases of diestrus, proestrus, estrus, and postestrus in the 8-week-old group. D1-D4 details the same phases in the 10-week-old group, while E1-E4 illustrates the phases in the 12-week-old group. Blue arrows represent neutrophils, red arrows reflect basal cells, and green arrows are keratinized cells. **(b)** The periodic changes in E2 levels in mice across the 4-week, 6-week, 8-week, 10-week, and 12-week age groups are shown (n = 4,mean ± SD),^*^P ＜ 0.05, ^**^P ＜ 0.01. **(c)** Representative HE staining images of the ovaries from mice of different ages (8 × , n = 3). Primordial follicles are marked by green arrows, primary follicles by red arrows, secondary follicles by yellow arrows, mature follicles by blue arrows, and the corpus luteum by blue stars.

To explore whether 8-week-old C57BL/6J mice are sexually mature, vaginal exfoliated cells from C57BL/6J mice of various ages were examined through HE staining. At sexual maturity, the exfoliated cells in the vagina were exhibited a high density of white blood cells during the diestrus phase, as well as round or oval basal cells and accessory basal cells. During proestrus, the number of round or oval basal cells and accessory basal cells increased and became predominant, with occasional keratinized cells observed. In estrus, numerous flat, polygonal keratinized cells with serrated edges and absent nuclei were present, while basal and accessory basal cells were scarce. In late estrus, keratinized cells gradually decreased, nucleated cells reappeared, and leukocytosis increased [22,23]. Results of the HE staining showed no differences in the vaginal cell smears from the 4-week and 6-week groups. Mice in the 4-week group were consistently in diestrus, while those in the 6-week group were predominantly in diestrus and proestrus. The vaginal cell smears of mice in the 8-week, 10-week, and 12-week groups revealed marked differences in the characteristic cell and clear estrous cycles (Fig. 3a).

To verify whether E2 fluctuations were regulator in C57BL/6j mice, serum samples were collected for E2 level measurement based on the results of vaginal smear analysis. Once a stable estrus cycle was achieved, E2 levels increased rapidly, reaching their peak during estrus. Next, a sharp decline from this peak was observed, which slowed after entering late estrus, reaching the lowest point during diestrus. Moreover, it was observed that mice in the 4-week and 6-week groups did not enter estrus, and no significant changes in E2 levels were observed between cycles. In contrast, the E2 levels in the 8 and 12-week groups were significantly increased during proestrus, demonstrating a significant and rapid upward trend (P < 0.05, Fig 3b), although the E2 level in the proestrus phase of the 10-week group did not show a significant difference, an obvious upward trend was still observable.

To clarify the variations in ovarian morphology and function among the groups, HE staining was performed on ovaries samples from C57BL/6j mice across the groups. Further analysis revealed that at 4 and 6 weeks of age, the ovaries were small and flat, predominantly containing primordial and primary follicles, with no discernible corpus luteum. However, by 8, 10, and 12 weeks of age, the ovaries were significantly enlarged and were full, presenting reduced number of secondary follicles. Mature follicles were observed in the ovaries of mice at 8 and 12 weeks, with distinct corpus luteum formation. Based on these findings, we infer that diverse physiological indicators of sexual maturation are presented in the 8-week-old female C57BL/6j mice (Fig. 3c).

In conclusion, female C57BL/6J mice reach sexual maturity at 8 weeks of age, and their estrous cycle, estrogen fluctuation, and ovarian structure all tend to be stable and mature during this period.

## Experiment on the modeling time of PMOP in 8-week-old female C57BL/6J mice

### Materials and methods

#### Experimental animals.

Twelve 8-week-old female C57BL/6J mice were purchased from Hunan Slaike Jingda Laboratory Animal Co., LTD., with the license number SYXK (Xiang) 2019–0009. Subsequently, the mice were housed in cases, with three mice per cage, while provided with unlimited access to water and a standard diet. The room temperature was maintained between 24–26 °C, with humidity levels set at 55%−60% and adjusted to adequate light/dark cycle. All mice were kept in a laboratory with SPF standards. Each mouse received an equal amount of the basic diet and was provided sufficient drinking water. They were allowed to acclimatize for one week and their body weight and physical examinations were conducted. The experiment was approved by the the Animal Ethics Committee of Hunan University of Traditional Chinese Medicine, and all animal care and experimental procedures conformed to the Guiding Opinions on the Good Treatment of Laboratory Animals issued by the Ministry of Science and Technology of the People’s Republic of China.

#### Main reagents and instruments.

The following reagents and instruments were utilized in the experiments: Isoflurane (Shenzhen Ruiwade Life Technology Co., LTD.); 4% paraformaldehyde; Penicillin sodium (North China Pharmaceutical Group Co., LTD., China); hematoxylin and eosin (HE) dye; BALP ELISA kit (Meimian, China, Item No.MM-44680M2); TRACP-5b ELISA kit (Meimian, China, Item No.MM-0408M2); Rayward R500 general respiratory anesthesia machine (Shenzhen Rayward Life Technology Co., LTD., China); Quantum FX Micro-CT (Perkin Elmer, USA); and desktop microfreezer centrifuge (Thermo, China).

#### Grouping of animals.

Mice weighing between 18-22g, without discernable abnormalities or diseases, were randomly divided into four groups using a standard random number table method: the sham group, the 6-week model group, the 9-week model group, and the 12-week model group, each with three animals. Those in the model groups were castrated to establish a model of postmenopausal osteoporosis, while those in the sham group underwent only superficial incisions to remove the epidermis, peritoneum, and a small portion of fat surrounding the ovary.

#### Modeling method.

The mice were subjected to one week of adaptive feeding, and then subjected to ovariectomy (OVX). Briefly, the mice were placed in an induction chamber pre-filled with isoflurane vapor and anesthesia was induced with 5% isoflurane delivered in oxygen at a flow rate of 1 L/min, until the righting reflex was lost. Subsequently, they were secured on a thermostatic surgical table and maintained under 2% isoflurane delivered via nose cone with 100% oxygen at a flow rate of 0.5 L/min. During the procedure, the anesthetic depth was monitored by toe pinch reflex and respiratory rate, during which the isoflurane concentration was adjusted if necessary. Following deep anesthesia, the superficial hair on the middle and lower third of the back was removed, and the area was disinfected using iodophor followed by 75% alcohol. A longitudinal incision was made at the surgical site, that was 1 cm below the rib margin and 1 cm lateral to the spine, to facilitate efficient separation of the skin, muscles, and peritoneum. Using sterile forceps, a blunt dissection was conducted along the midline of the back to both sides of the spine. Once the white adipose tissue beneath the peritoneum was exposed, one side of the peritoneum was incised, granting access to the abdominal cavity. The adipose layer was retracted to locate the uterus, from which one uterine horn was gently extracted, revealing the ovary at its end. The ovary appeared pink, had a mulberry shape and was encased in white adipose tissue. A hemostat was utilized to clamp the root of the ovary, after which the fallopian tube along with the accompanying blood vessels were ligated using 4−0 Vicryl sutures. Both ovaries were entirely excised, to ensure that the removed tissue contained ovarian parenchyma. Hemostasis was performed, the uterus and fallopian tubes were placed back into the abdominal cavity, and the peritoneum, muscles, and skin were sutured sequentially in layers. Postoperatively, carprofen (5 mg/kg) was administered subcutaneously to control pain for three days postoperatively. Penicillin was administered to prevent infection [24,25]. In the sham group, an incision was made on the peritoneum to expose the adipose tissue adjacent to the ovary. Then, an equivalent in volume to the ovary (approximately 3 × 3 mm), was excised, and the remaining procedures were similar to those seen in the OVX group to ensure consistency in surgical trauma. Postoperative care included provision of soft bedding and thermal insulation measures.The mice were monitored daily to examine the wound healing process, activity levels, and food/water intake. Additional anti-inflammatory or analgesic treatment was administered if signs of infection or pain (e.g., lethargy, piloerection) were observed. Weekly weight assessments and change of cages every three days were also performed.

#### Indicators of observation.

(1) Vaginal exfoliated cell smear

On the third day after operation, mice from the sham and model groups were selected at 9:00 AM. We then administered 10 µL of normal saline into the vaginal cavity of the mice using a 0.5–10 µL pipette, about 5–6 times. The vaginal secretion was obtained on an adhesive slide, and a cover slip was employed to ensure uniform spread of the secretion. The slides were allowed to air dry and then stained with HE reagent once daily for seven consecutive days.

(2) Morphological observation of mouse uterus

Moreover, changes in body weight of the mice were recorded at 9:00 AM on the day of modeling, and subsequently on a weekly basis at the same 9:00 AM. The final body weights were recorded at the 6th, 9th, and 12th weeks post-modeling. The mice were placed on the supine position on the operating table, and the skin and muscle layers were incised along the midline of the abdomen using scissors to expose the abdominal organs. The uterus, characterized by a “Y”-shaped structure located in the lower abdominal cavity, was identified, and the cervix was gently elevated using forceps, and the junction between the cervix and vagina was carefully severed.

(3) Observation of bone histomorphology

At 6-, 9-, and 12-weeks post-modeling, mice in the model groups corresponding to these time points were sacrificed via cervical dislocation, while those in the sham group were euthanized 6 weeks post-modeling. The left femurs from three mice per group were obtained and prepared for HE staining. The femur was extracted and the surrounding muscle as well as soft tissues were carefully removed using gauze, and the femur was then fixed via immersion in 4% paraformaldehyde. After 24 hours of fixation, the samples were rinsed with phosphate-buffered saline (PBS) and decalcified in 10% EDTA at pH 7.4 at 4°C, with fresh solutions being replaced daily. Subsequently, the bone tissue was gently probed with a needle tip until no resistance was detected. It was then dehydrated, subjected to transparency treatment, embedded, sectioned, and stained with HE to determine the changes in bone microstructure [26].

(4) Micro-CT

The right femurs of three mice from each group were subjected to Micro-CT scanning. Next, the mice were sacrificed via cervical dislocation, the femur specimens, which were devoid of surrounding soft tissue, were immersed in 4% paraformaldehyde for fixation before Micro-CT scanning. The scanning parameters were set as follows: source voltage at 70 kV, source current at 130 μA, exposure time of 240 ms, resolution of 10 μm, and a 0.2 mm thickness aluminum filter. The distal femur of the mouse was targeted in the scanning. The bone mineral density (BMD), bone volume fraction (BV/TV, %), trabecular number (Tb.N, 1/nm), and trabecular separation (Tb.Sp, μm) of the region 1 mm to 2 mm from the distal femoral epiphyseal plate were finally calculated.

(5) Detection of bone metabolism

Indexes Orbital blood samples were collected at 6-, 9-, and 12-weeks post-modeling, centrifuged at 1500 r/min with a radius of 7 cm at 4°C for 20 minutes. The supernatant was obtained and subjected to ELISA to quantify the levels of TRACP and B-ALP.

#### Statistical methods.

All experimental data were subjected to normality tests. Data that conformed to a normal distribution were expressed as mean ± standard deviation (mean ± SD).The Student’s t-test was employed to compare two groups while multiple groups were compared using the one-way analysis of variance (one-way ANOVA. If the ANOVA results showed significant differences, Tukey’s post-hoc test was further used for pairwise comparisons. For data that did not conform to a normal distribution, the Mann-Whitney U test was utilized to compare two groups while the Kruskal-Wallis test was applied in multiple groups comparisons. If there were overall differences, Dunn’s test was used for post-hoc analysis. All statistical analyses were performed using Graphpad Prism 10 software, and the threshold for statistical significance was set at P < 0.05.

### Result

**Fig 4:**
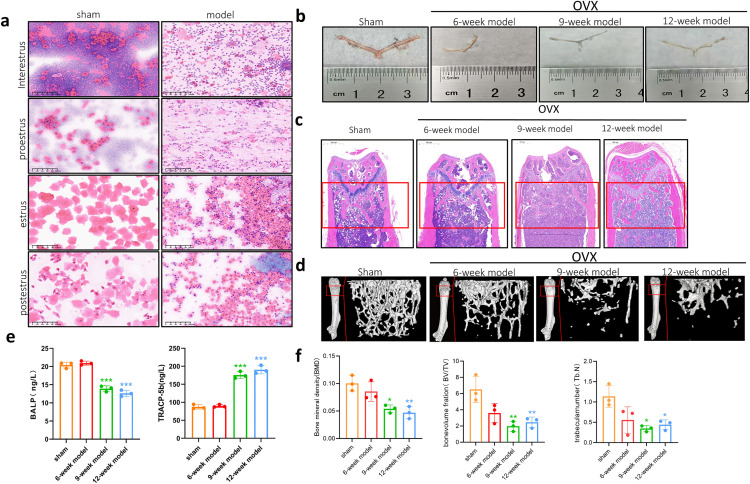
Modeling results of 8-week-old female C57BL/6J mice. **(a)** Comparison of postoperative vaginal exfoliated cell smears between the sham group and model group (20 × , n = 3). **(b)** Comparison of uterine appearances in the indicated groups. A represents the uterus of the sham operation group, B corresponds to the 6-week model group, C to the 9-week model group, and D to the 12-week model group. **(c)** Representative HE staining images of distal femurs in each group (3 × , n = 3). **(d)** Representative Micro-CT three-dimensional reconstruction images in the indicated groups (n = 3). **(e)** Changes in serum levels of bone metabolism markers across each group (n = 3, mean ± SD). Compared with the sham group, ^***^P < 0.001 **(f)** Quantitative analysis of BMD, BV/TV and Tb.N in the indicated groups (n = 3, mean ± SD). Compared with the sham group, ^*^P < 0.05, ^**^P < 0.01.

Mice in the sham group exhibited complete and regular estrous cycle, whereas the model group showed disruptions during both the interestrus and proestrus phases. In the sham-operated group, proestrus was characterized by a prominent presence of basal and accessory basal cells, along with noticeable lymphocyte infiltration. Pronounced pink sheets of keratinized cells lacking nuclei were detected during estrus. In the anaphase, basal cells presented a mixed state of nucleated round cells and non-nucleated sheet cells. During the diestrus phase, cytopenia and extensive inflammatory cell infiltration were observed. In the model group, significant nucleated basal cells and accessory basal cells were observed, accompanied with marked disruption of the estrous cycle during both estrus and proestrus (Fig 4a).

To clarify the success of oophorectomy and uncover the effects of E2 deficiency on uterine morphology, we collected samples at 6-, 9-, and 12-weeks post-operation. Mice in the sham group exhibited a well-defined uterine shape, a thick uterine body, and a fleshy pink coloration. In contrast, the uterine morphology of mice subjected to bilateral ovariectomy was significantly atrophic, irregular in shape, reduced in size, and demonstrated a gray coloration (Fig 4b).

Considering that the timing for establishing a model of postmenopausal osteoporosis due to E2 deficiency-induced bone loss has not been sufficiently studied, we performed a more comprehensive understanding of trabecular bone loss, bone microarchitecture, and osteocytic metabolism at 6, 9, and 12 weeks post-surgery. The HE staining, Micro-CT three-dimensional reconstruction of the femur, and detection of bone metabolic markers were performed at each time-point. Results of the HE staining demonstrated that the distal femoral trabeculae in the sham group were compact, complete, and continuous. Over time, the trabecular bone in the model groups progressively became disorganized, fragmentated, and reduced. Notably, the degree of trabecular bone fracture disorder in mice from the 9-week and 12-week model groups were significantly increased, with the most pronounced changes observed. However, there was no significant difference in trabecular bone changes between the 9-week and 12-week model groups (Fig 4c). Micro-CT analysis revealed that the sham-operated group had dense, abundant trabeculae and an intact bone microstructure. In contrast, the model group showed a progressive reduction in trabecular bone and gradual deterioration of the bone microstructure at 6-, 9-, and 12-weeks post-operation. Notably, the alterations in bone trabeculae and microstructure in the model groups were more pronounced at the 9- and 12-week marks (Fig 4d). The BMD, BV/TV, and Tb.N were comparable between the 6-week model group and the sham group. In contrast, the BMD, BV/TV, and Tb.N were significantly lower in the 9-week and 12-week model groups relative to levels in the sham group (*P* < 0.05, *P* < 0.01). The 12-week model group presented a modest, non-statistically significant decrease in BMD, BV/TV, and Tb.N compared to the 9-week model group (Fig 4f). Bone metabolism marker analysis revealed that the levels of bone-specific alkaline phosphatase (BALP) and tartrate-resistant acid phosphatase 5b (TRACP-5b) in the 6-week model group were comparable to those in the sham group. However, BALP levels in the 9-week and 12-week model groups were significantly lower relative to those in the sham group. Conversely, circulating TRACP-5b levels were significantly elevated (*P* < 0.001, Fig 4e).

Studies have confirmed that the model established by ovariectomy can effectively simulate a series of physiological changes caused by estrogen deficiency, including reproductive cycle disorders, uterine degeneration, as well as significant bone loss and deterioration of bone microstructure observed 9 weeks after surgery.

## Discussion

Postmenopausal osteoporosis (PMOP) is a major metabolic disease that manifests as the functional imbalance between osteoclasts and osteoblasts following E2 deficiency. Its primary pathological features include decreased bone mass and bone microstructure damage [27,28]. The rising aging population has increased the incidence of PMOP, now accounting for 80% of all individuals with osteoporosis, making it a major public health concern that impacts women’s health and quality of life [29,30]. This calls for development of standardized animal models to facilitate future investigations into the pathological mechanisms and drug interventions associated with PMOP. However, there are no unified standards for selecting appropriate species, the age of animals at the time of surgery, and the postoperative observation time points for bone loss [31,32]. This inconsistency creates high heterogeneity in the available experimental results, which increases the difficulty of accurately reflecting the disease progression. Here, we integrated bibliometric analysis with multi-time point experimental verifications to not only demonstrate the sexual maturity characteristics of C57BL/6J mice and the patterns of bone loss following OVX, but also reveal that the ninth week post-OVX is a window of phenotypic stability. This finding may serve as a reference for optimizing PMOP animal models in basic research.

To reduce the reliance on empirical judgment and minimize the subjectivity associated with traditional modeling approaches, we applied bibliometric analysis to quantitatively evaluate key features of the academic literature, including publication volume, authorship, institutional affiliations, keyword trends, and citation networks. This method provides a more objective and data-driven perspective on the development and application of PMOP models [33]. This study employed bibliometric methods based on a decade’s literature analysis from the Web of Science database, and found that C57BL/6J mice accounted for 44.3% of PMOP models, which exceeded that of other species, such as SD rats and BALB/c mice. Notably, 8-week-old C57BL/6J mice were recognized as the most frequently utilized surgical model.This widespread application not only reflects its general acceptance within the research community but also stems from a deeper reason: as an inbred strain, C57BL/6J mice possess the unique advantages of a well-defined genetic background and stable phenotypes. These characteristics are crucial for ensuring the consistency of bone loss responses after OVX and the comparability of experimental results [34,35]. However, bibliometric analysis has certain limitations. The results obtained by this method reflect what is ‘most commonly used’ rather than what is ‘optimal.’ High-frequency usage may be influenced by historical inertia, availability of animal models, cost, and other factors, potentially overlooking new methods or unpublished data, suggesting that the available data are not comprehensive. Therefore, further conducted biological validation and model efficacy evaluation experiments using the 8-week-old C57BL/6J mice.

Studies have shown that vaginal exfoliated cell smears, E2 levels, and ovarian histological analysis are core indicators for evaluating the sexual maturation status of experimental animals [36–39]. The estrous cycle of mice lasts 4–5 days and includes proestrus, estrus, postestrus, and interestrus phases. The cellular composition varies at each stage, reflecting cyclical fluctuations of sex hormones [40]. Here, we found that the vaginal smears of C57BL/6J mice aged 8–12 weeks exhibited regular estrous cycles, but there no significant periodic changes in the 4-week and 6-week groups. This suggests that 8 weeks of age provides a stable window for examining sexual maturation. As a direct marker of E2 activity, the periodic fluctuations in serum E2 levels influence ovarian function [41]. These findings illustrated that the E2 levels in proestrus for 8–12-week-old mice were significantly higher relative to those in interestrus (P < 0.05), and no significant fluctuations in E2 levels were detected in the 4-week and 6-week groups. This further supported the notion that 8 weeks of age is the appropriate time for surgical intervention. Mature ovaries contain mature follicles and corpus luteum [42,43]. Histological analyses indicated that 8–12 week old mice exhibited full ovarian volume and mature follicular and corpus luteum structures, while 4-week and 6-week old mice display flat ovaries with a predominance of primordial follicles. Therefore, we conclude that, the 8-week-old C57BL/6J mice are sexually mature, making them an ideal choice for developing a PMOP model due to the optimal balance between feeding cycle, cost, and postoperative recovery ability when compared to older mice.Compared with rats or other outbred mouse strains, C57BL/6J mice have comprehensive advantages in genetic consistency, sensitivity to bone loss, and economic feasibility. These advantages further support the selection of this strain in the present study as well as in most studies within the field [44].

Analysis of vaginal exfoliated cells following OVX may display the fluctuations in sex hormones in animals. The uterus serves as a classical target organ for E2, and its morphological changes are among the most intuitive indicators for evaluating ovarian failure. These makes them ideal criteria for assessing the success of OVX [45]. This study revealed that vaginal smears post-OVX surgery were predominantly composed of leukocytes, basal cells, and parabasal cells, without a discernable estrus. Furthermore, significant uterine atrophy and thinning were detected in the model groups at 6, 9, and 12 weeks. These direct phenotypic changes confirmed the successful removal of the ovaries and the effective reduction in E2 levels, mirroring the features of postmenopausal estrogen-deficient state. In recent years, advancements in imaging technologies, particularly the application of three-dimensional mapping in cardiac pacing [46], have revealed important insights that can be used to monitor and evaluate of animal models. In the present investigation, Micro-CT technology in conjunction with histological observation to dynamically monitor changes in bone microstructure in a postmenopausal osteoporosis model and examined the degree of bone loss based on parameters such as bone mineral density (BMD), bone volume fraction (BV/TV), and trabecular number (Tb.N) [47,48]. Moreover, bone metabolism markers BALP and TRACP-5b can help to evaluate the activity of osteoblasts and osteoclasts, and they also reflect the state of bone metabolism at various stages [49–51]. Histological and micro-CT analyses revealed that the model group progressively experienced bone loss and microstructural changes over time. However, no significant differences were observed between the 6-week model group and the sham group, nor were there notable fluctuations in BALP levels and TRACP-5b. At 9 and 12 weeks post-operation, trabecular bone fractures and disorder were noted in the model group, accompanied with elevated TRACP-5b levels, while BALP, BMD, BV/TV, and Tb.N were significantly decreased (*P* < 0.05, *P* < 0.01). However, modest, non-statistically significant decreases in BALP, BMD, BV/TV, and Tb.N were observed, accompanied with a slight, non-statistically significant increase in TRACP-5b in the 9-week model group compared with the 12-week model group. This suggests that bone loss was exacerbated and osteolysis was enhanced accelerated in the model at 9 weeks post-OVX surgery, with a transition to a sustained and gradual bone loss phase beginning thereafter. The absence of significant differences in these parameters between the 9-week and 12-week time points suggests that the most pronounced phase of trabecular bone perforation and structural deterioration likely occurred prior to or by the 9-week mark. Subsequently, slower thinning or sustained loss occurs within the already compromised structures. Therefore, the 9-week post-surgery period represents a critical window of stability for the OVX-induced PMOP phenotype, and the combined analysis of multiple assays provides a multi-dimensional scientific basis for model evaluation.

In this study, we combined bibliometrics and animal experiments, revealing that 8-week-old C57BL/6j mice are most widely used in the PMOP model. The features of sexual maturity meet the OVX modeling criteria, and evident postmenopausal osteoporosis phenotypes can be observed 9 weeks post-surgery, making them ideal mice for PMOP modeling. The present findings establish a standardized model and identify key time points for investigating the pathophysiological mechanisms of PMOP and evaluating potential therapeutic interventions. Nonetheless, there are certain limitations, including a relatively short observation period and the absence of detailed investigation into the specific mechanisms through which estrogen deficiency modulates bone metabolism. Future investigations should aim to extend the dynamic monitoring period post-surgery and integrate multi-omics technologies to analyze the dynamic regulatory network of bone metabolism post-OVX, thereby clarify the specific mechanisms by which E2 deficiency leads to osteoporosis.

## Supporting information

S1 File S1 Data. Bibliometric analysis. S2 Data. Estrogen level analysis. S3 Data. MicroCT quantification. S4 Data. Markers of bone metabolism. (ZIP)
