# Supplementary figures and images for: Determination of appropriate time for establishing a model of postmenopausal osteoporosis induced by bilateral oophorectomy: From bibliometric analysis to animal experiment
Source: PLoS One. 2025 Dec 4;20(12):e0336703. doi: 10.1371/journal.pone.0336703 (PMC12677515; doi:10.1371/journal.pone.0336703)

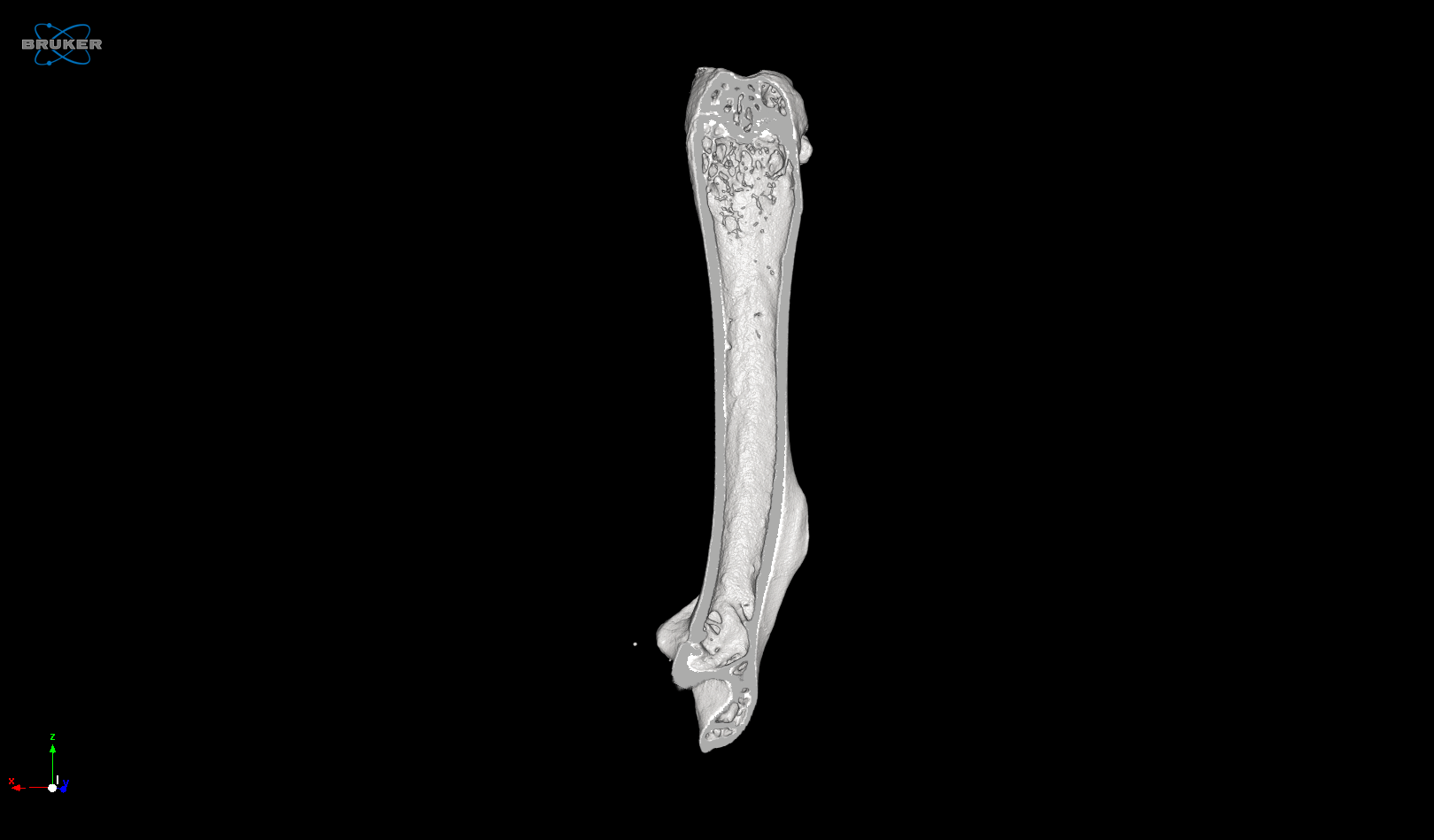

Supplement: S1 File — (ZIP) [file pone.0336703.s001.zip › Supporting Information Captions/S3_Data_MicroCT_quantification/Picture/12-week model/12week-1-1.bmp]

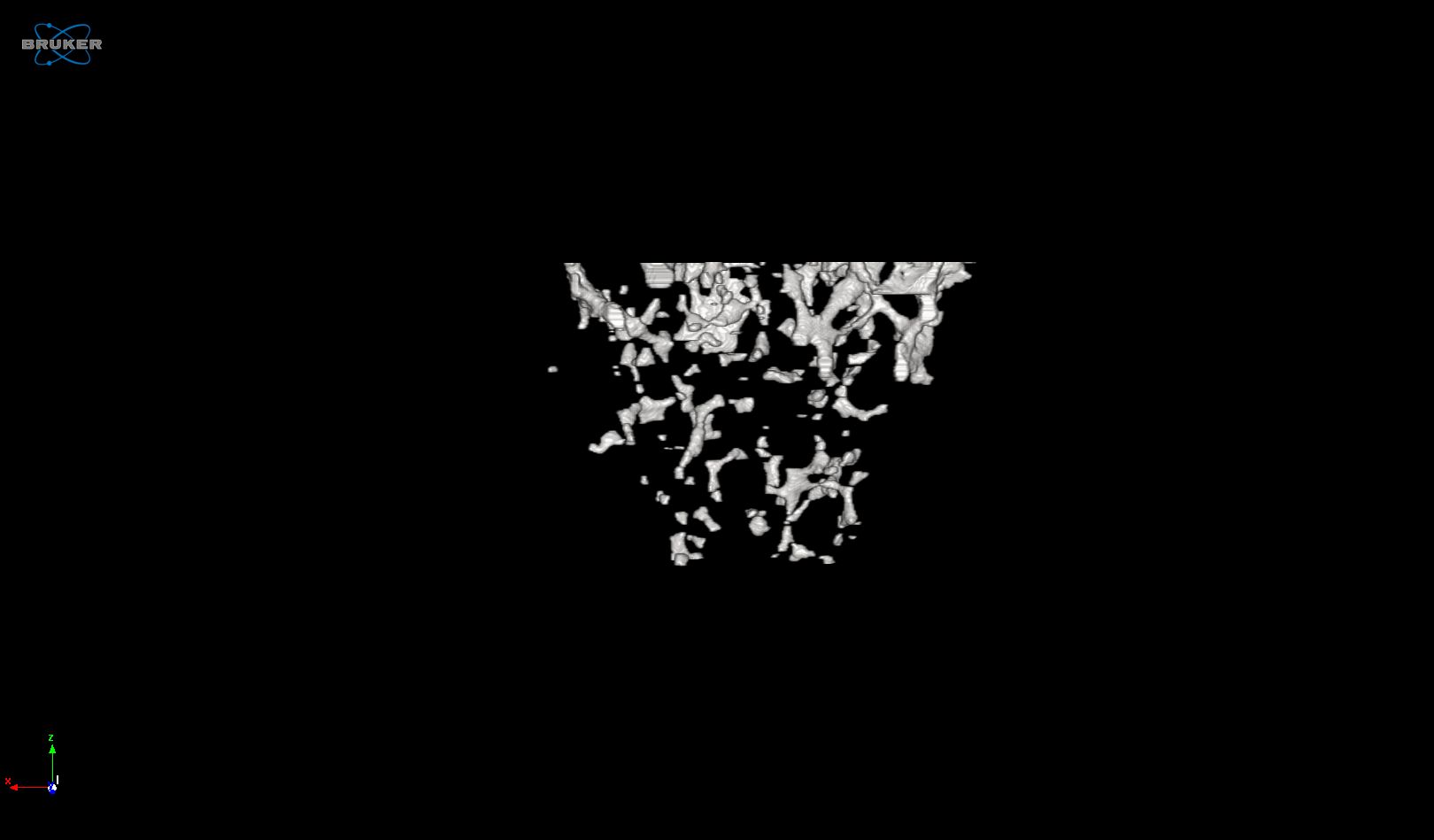

Supplement: S1 File — (ZIP) [file pone.0336703.s001.zip › Supporting Information Captions/S3_Data_MicroCT_quantification/Picture/12-week model/12week-1-2.bmp]

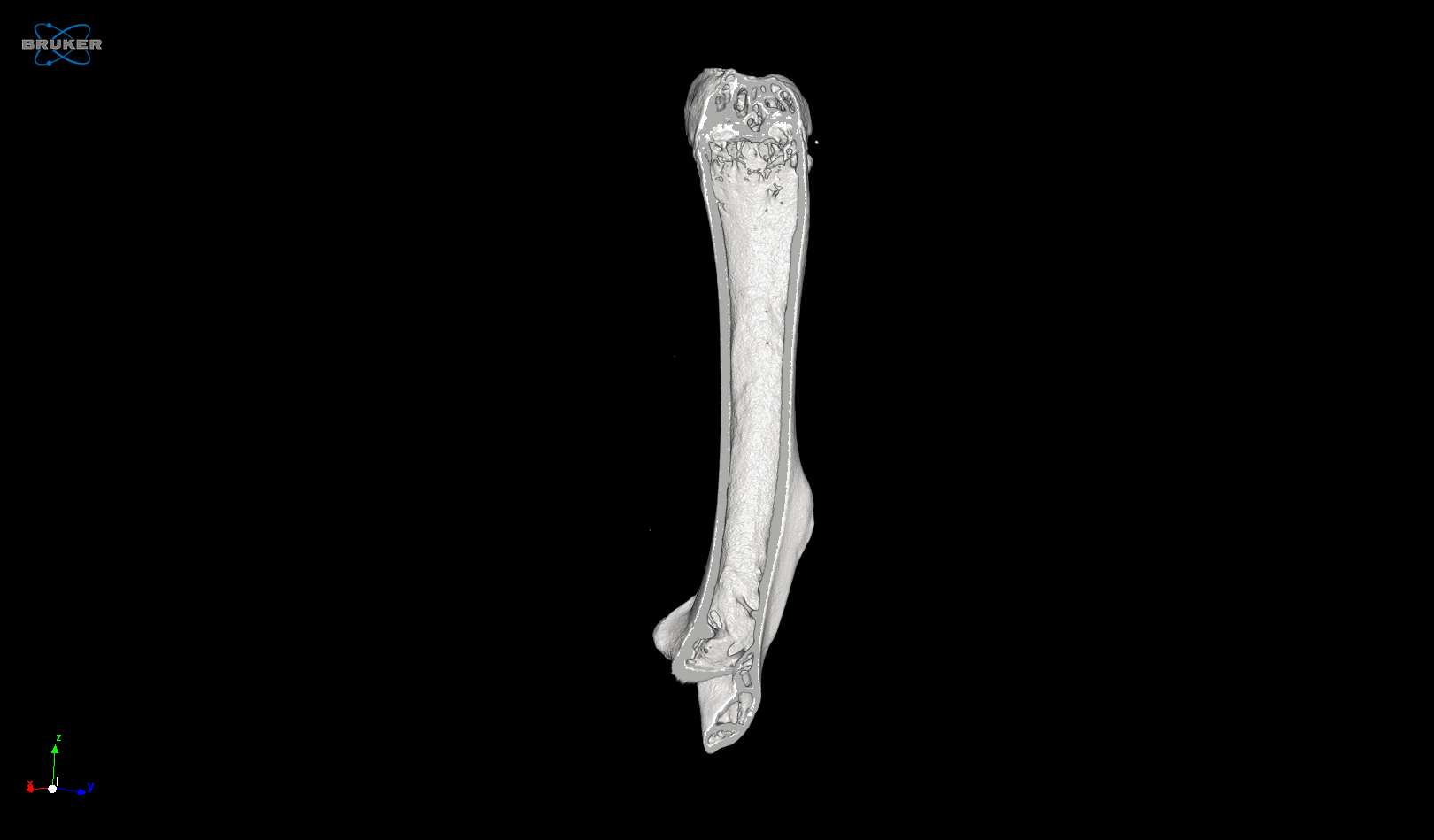

Supplement: S1 File — (ZIP) [file pone.0336703.s001.zip › Supporting Information Captions/S3_Data_MicroCT_quantification/Picture/12-week model/12week-2-1.bmp]

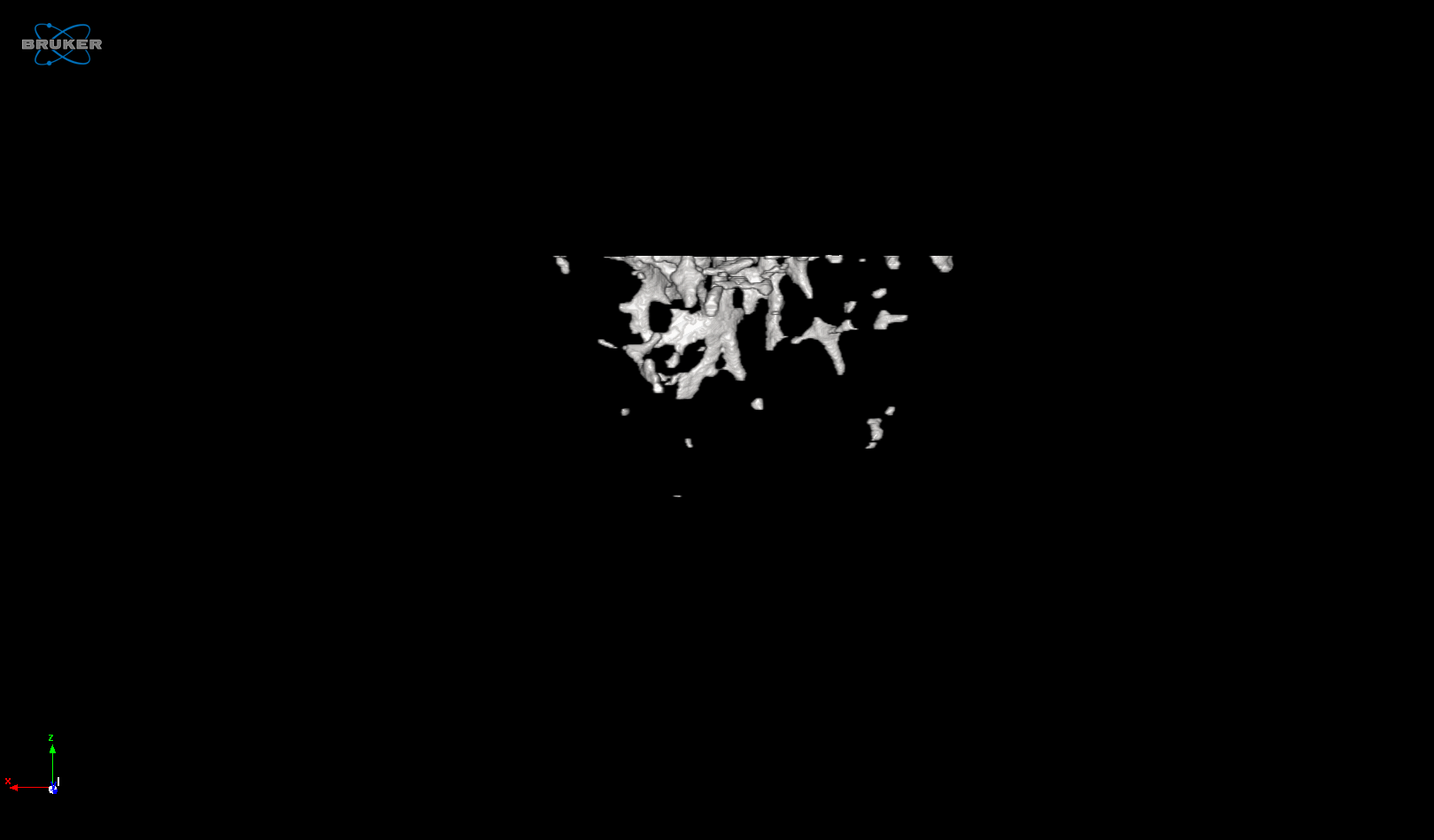

Supplement: S1 File — (ZIP) [file pone.0336703.s001.zip › Supporting Information Captions/S3_Data_MicroCT_quantification/Picture/12-week model/12week-2-2.bmp]

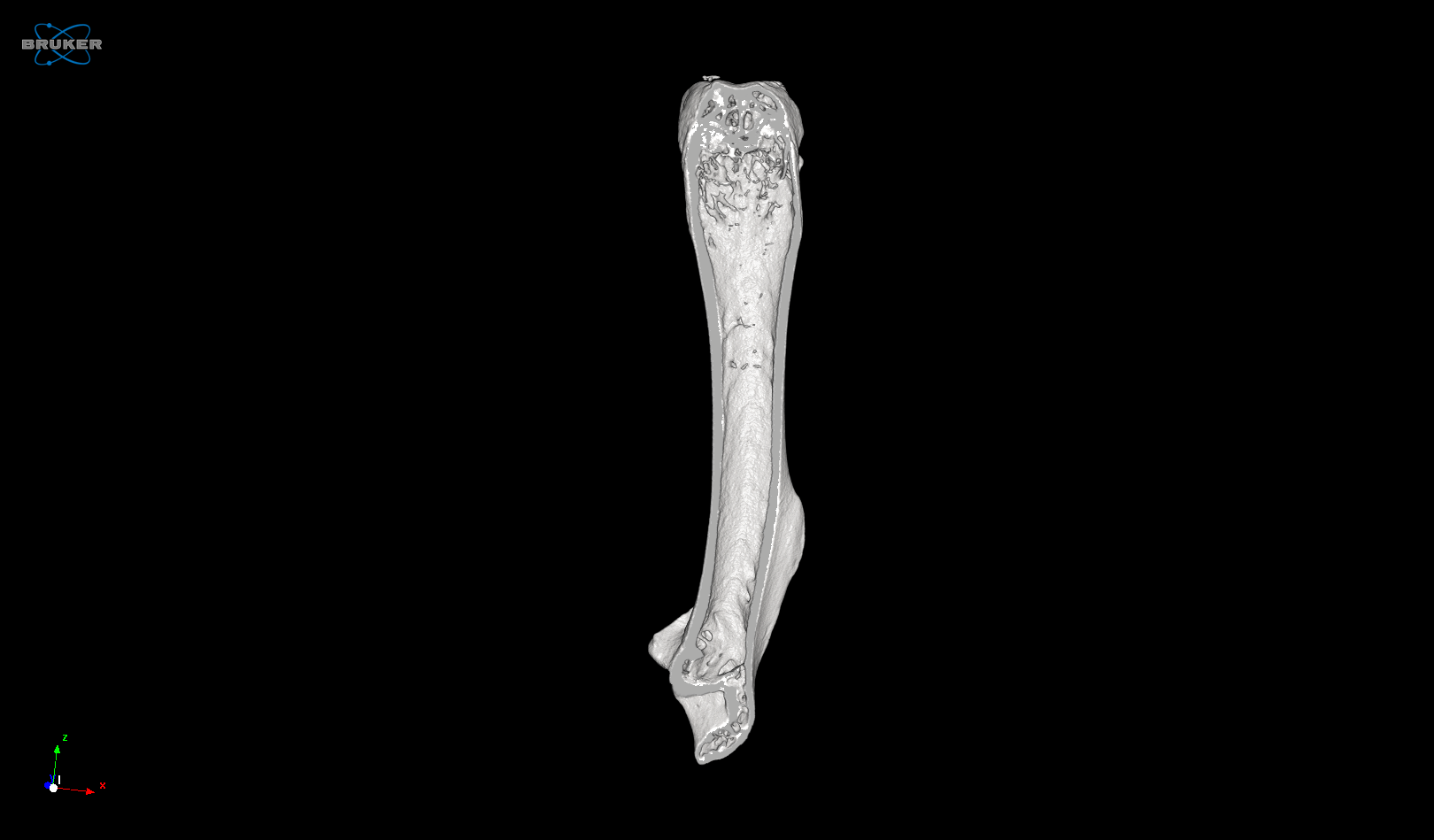

Supplement: S1 File — (ZIP) [file pone.0336703.s001.zip › Supporting Information Captions/S3_Data_MicroCT_quantification/Picture/12-week model/12week-3-1.bmp]

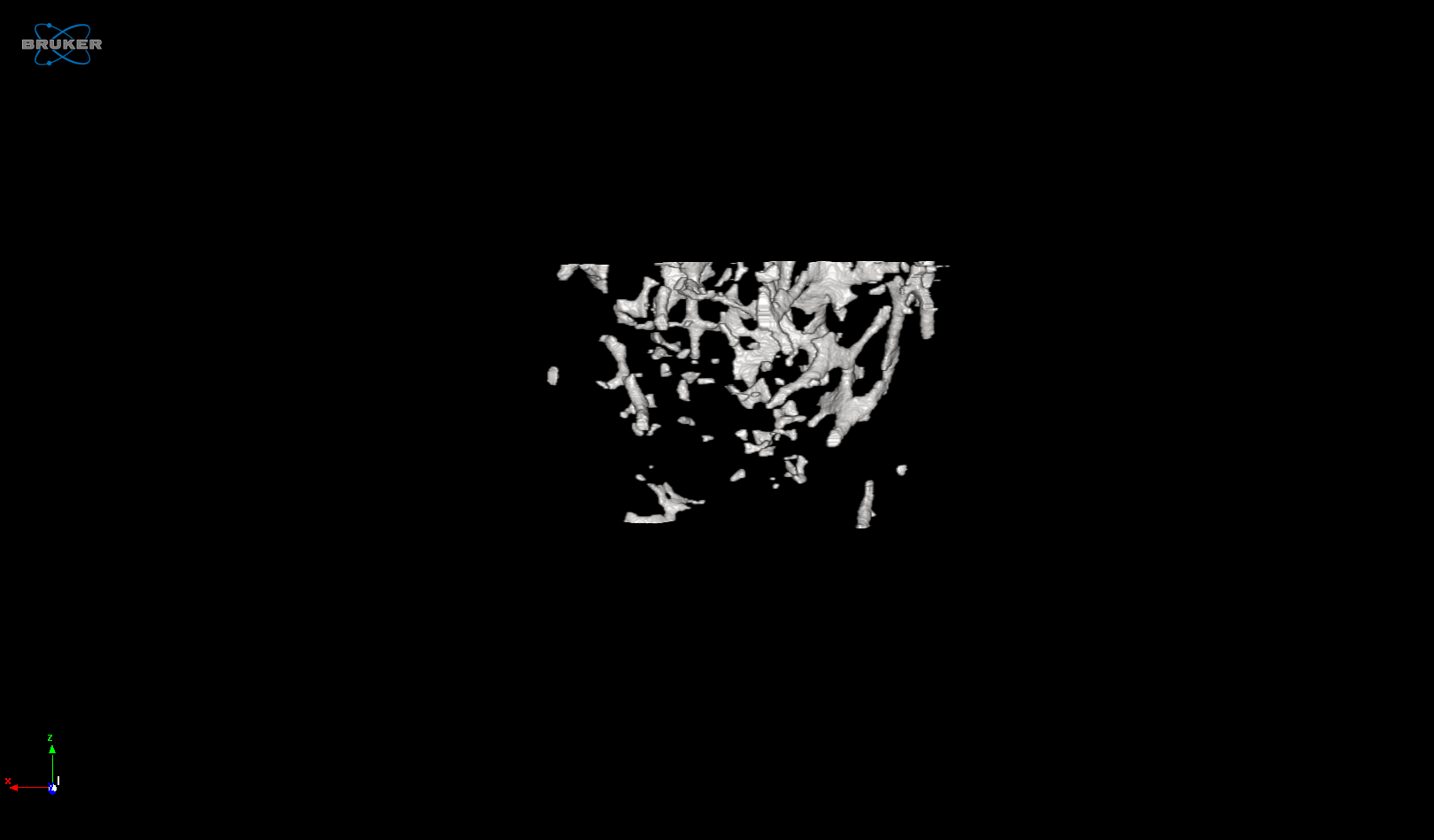

Supplement: S1 File — (ZIP) [file pone.0336703.s001.zip › Supporting Information Captions/S3_Data_MicroCT_quantification/Picture/12-week model/12week-3-2.bmp]

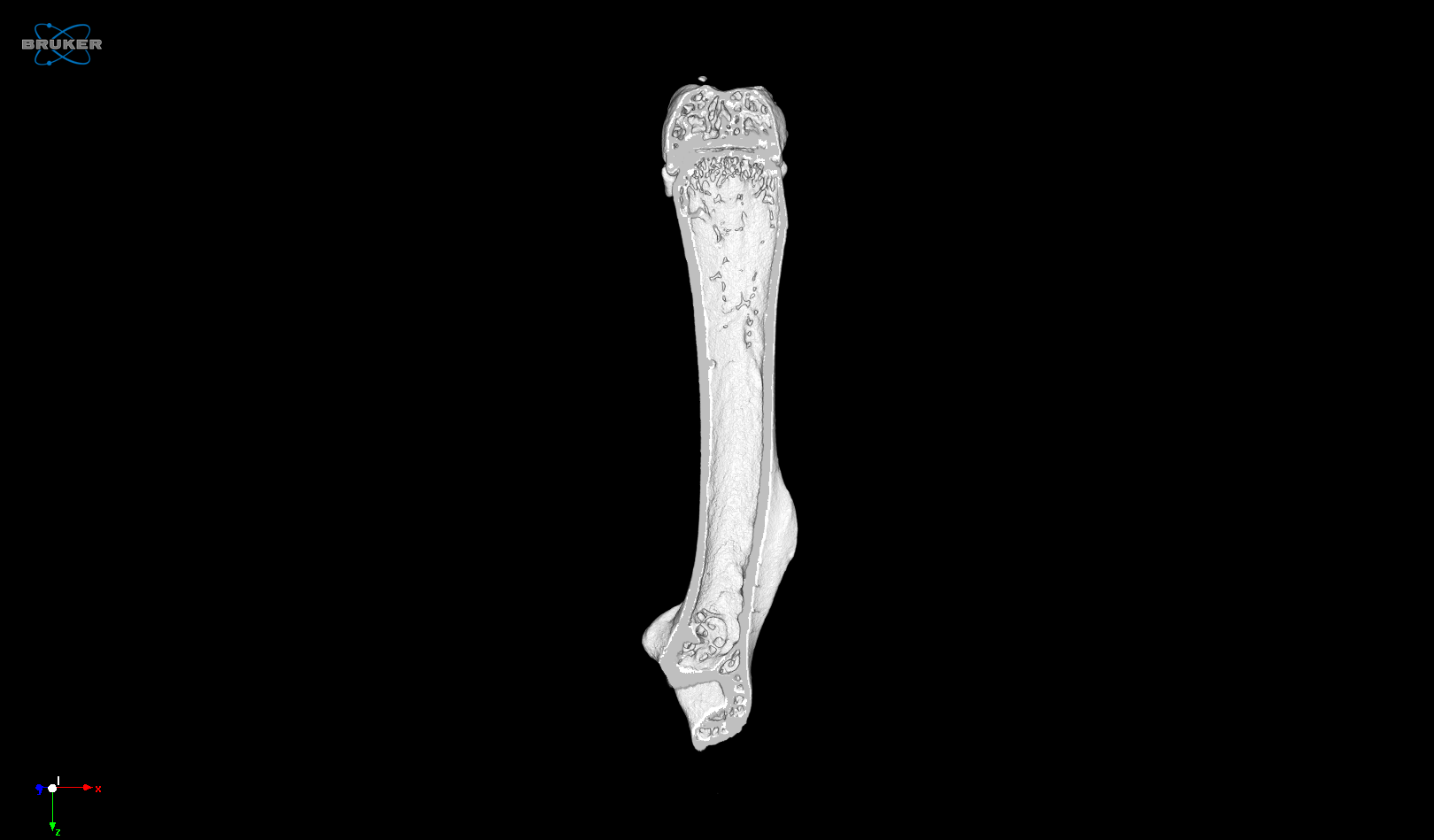

Supplement: S1 File — (ZIP) [file pone.0336703.s001.zip › Supporting Information Captions/S3_Data_MicroCT_quantification/Picture/6-week model/6week-1-1.bmp]

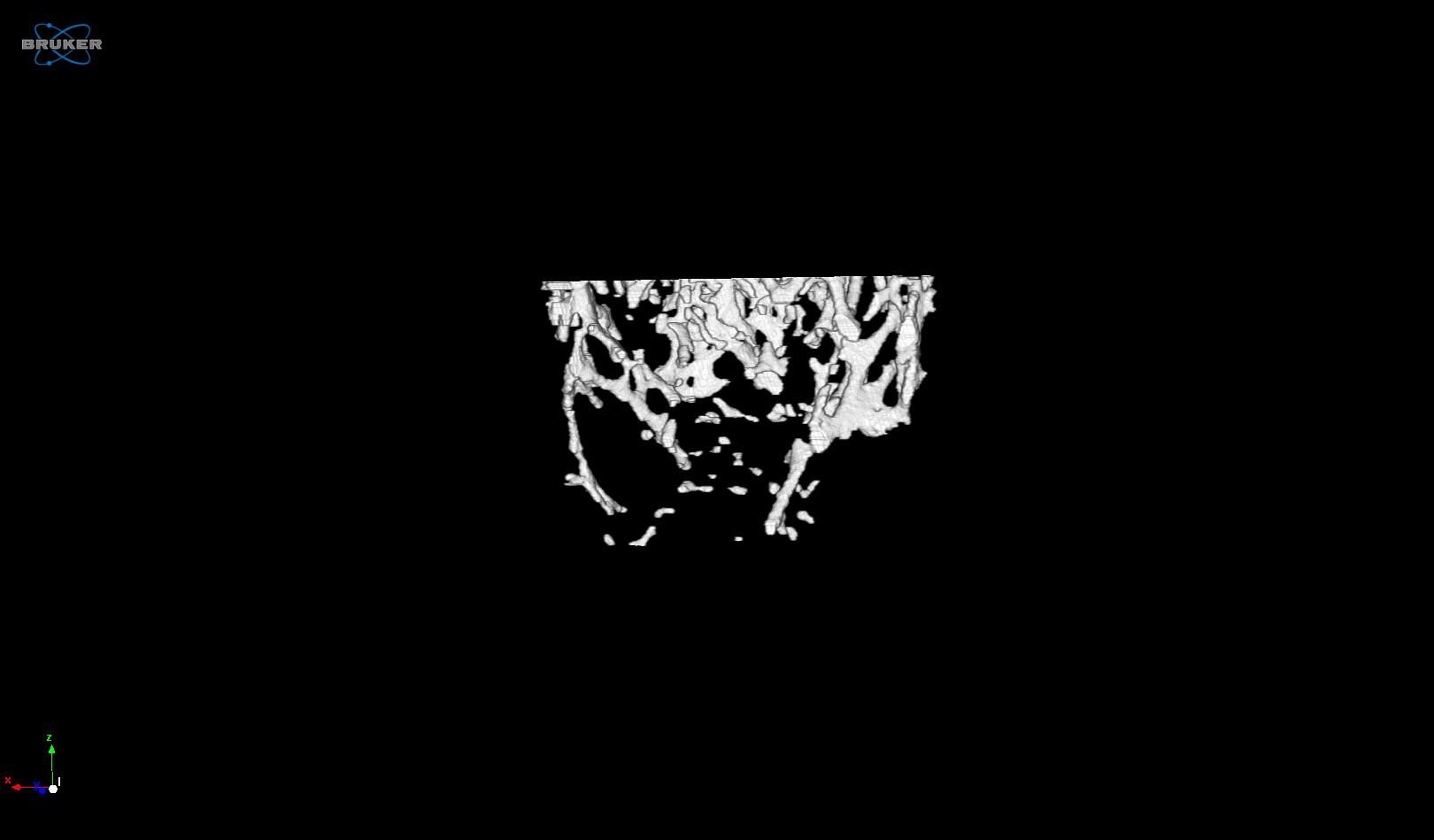

Supplement: S1 File — (ZIP) [file pone.0336703.s001.zip › Supporting Information Captions/S3_Data_MicroCT_quantification/Picture/6-week model/6week-1-2.bmp]

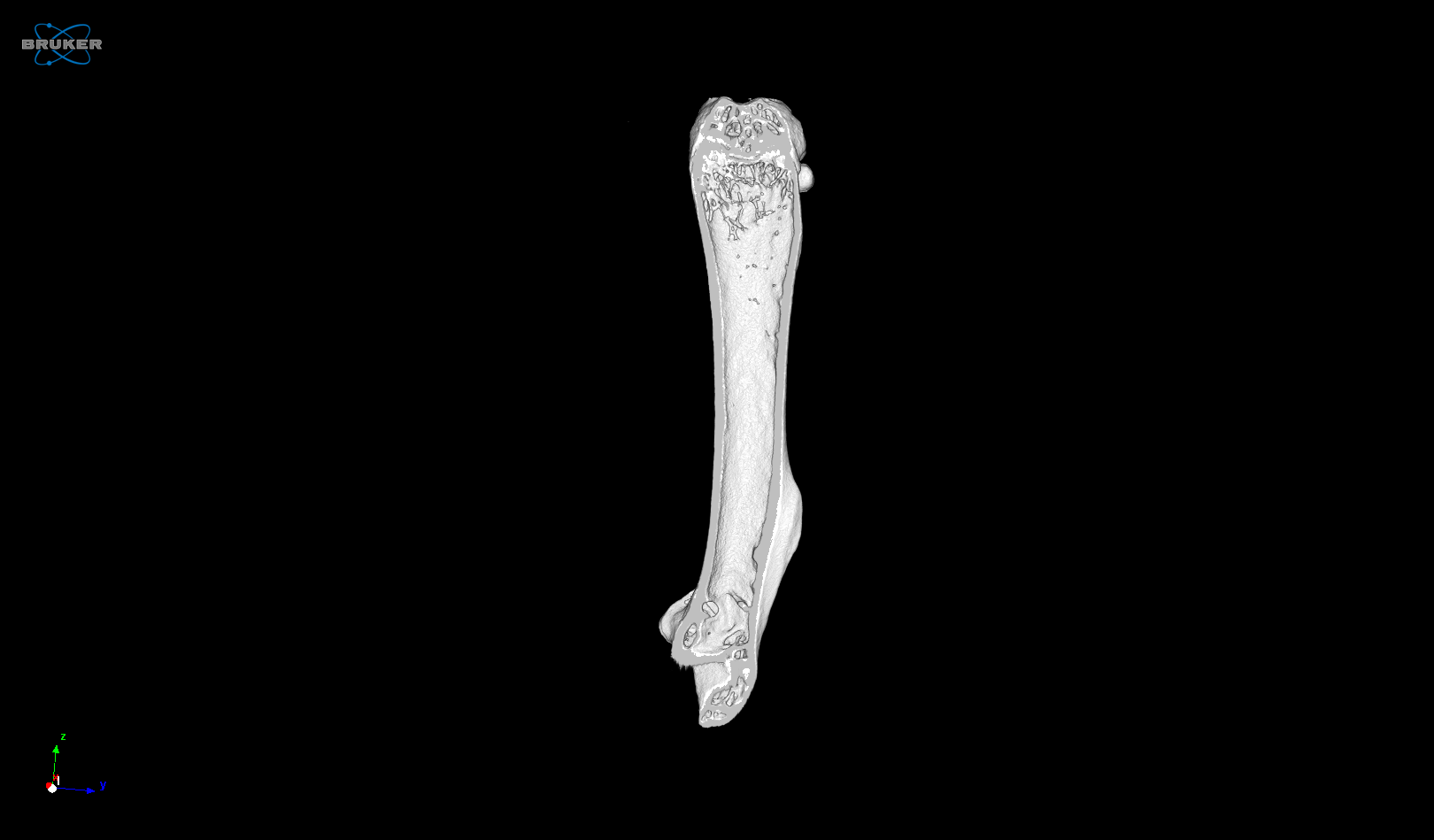

Supplement: S1 File — (ZIP) [file pone.0336703.s001.zip › Supporting Information Captions/S3_Data_MicroCT_quantification/Picture/6-week model/6week-2-1.bmp]

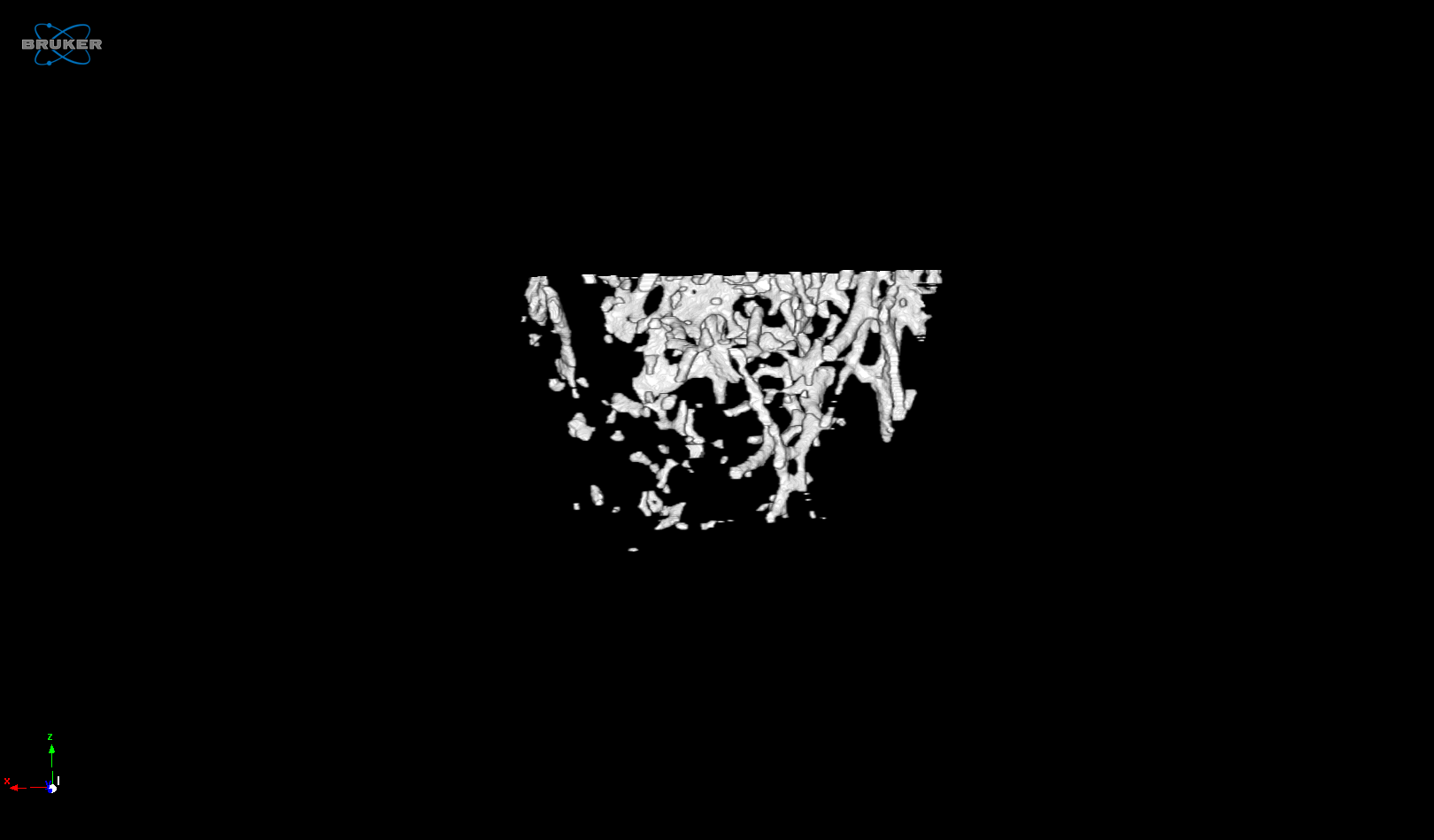

Supplement: S1 File — (ZIP) [file pone.0336703.s001.zip › Supporting Information Captions/S3_Data_MicroCT_quantification/Picture/6-week model/6week-2-2.bmp]

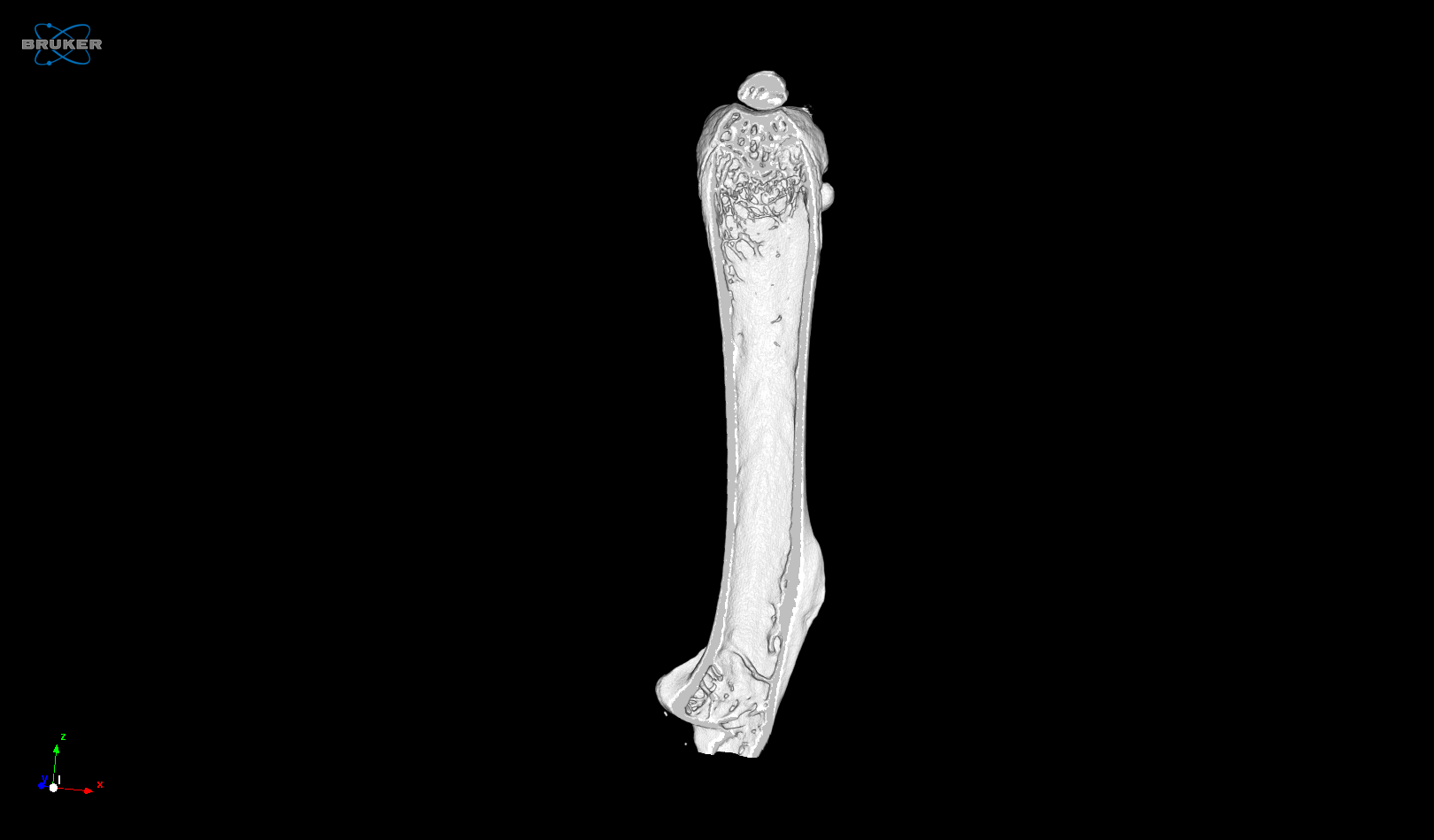

Supplement: S1 File — (ZIP) [file pone.0336703.s001.zip › Supporting Information Captions/S3_Data_MicroCT_quantification/Picture/6-week model/6week-3-1.bmp]

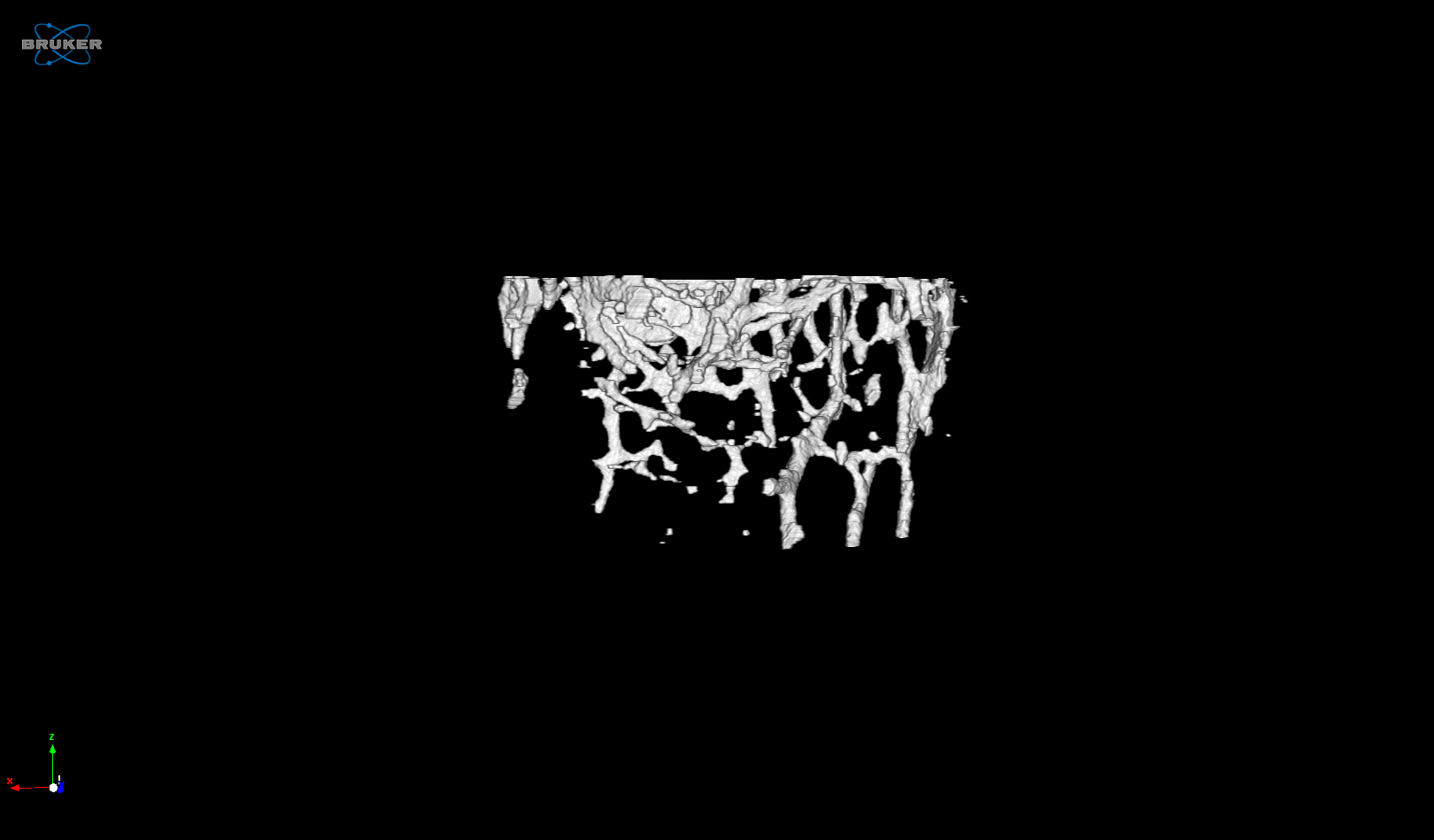

Supplement: S1 File — (ZIP) [file pone.0336703.s001.zip › Supporting Information Captions/S3_Data_MicroCT_quantification/Picture/6-week model/6week-3-2.bmp]

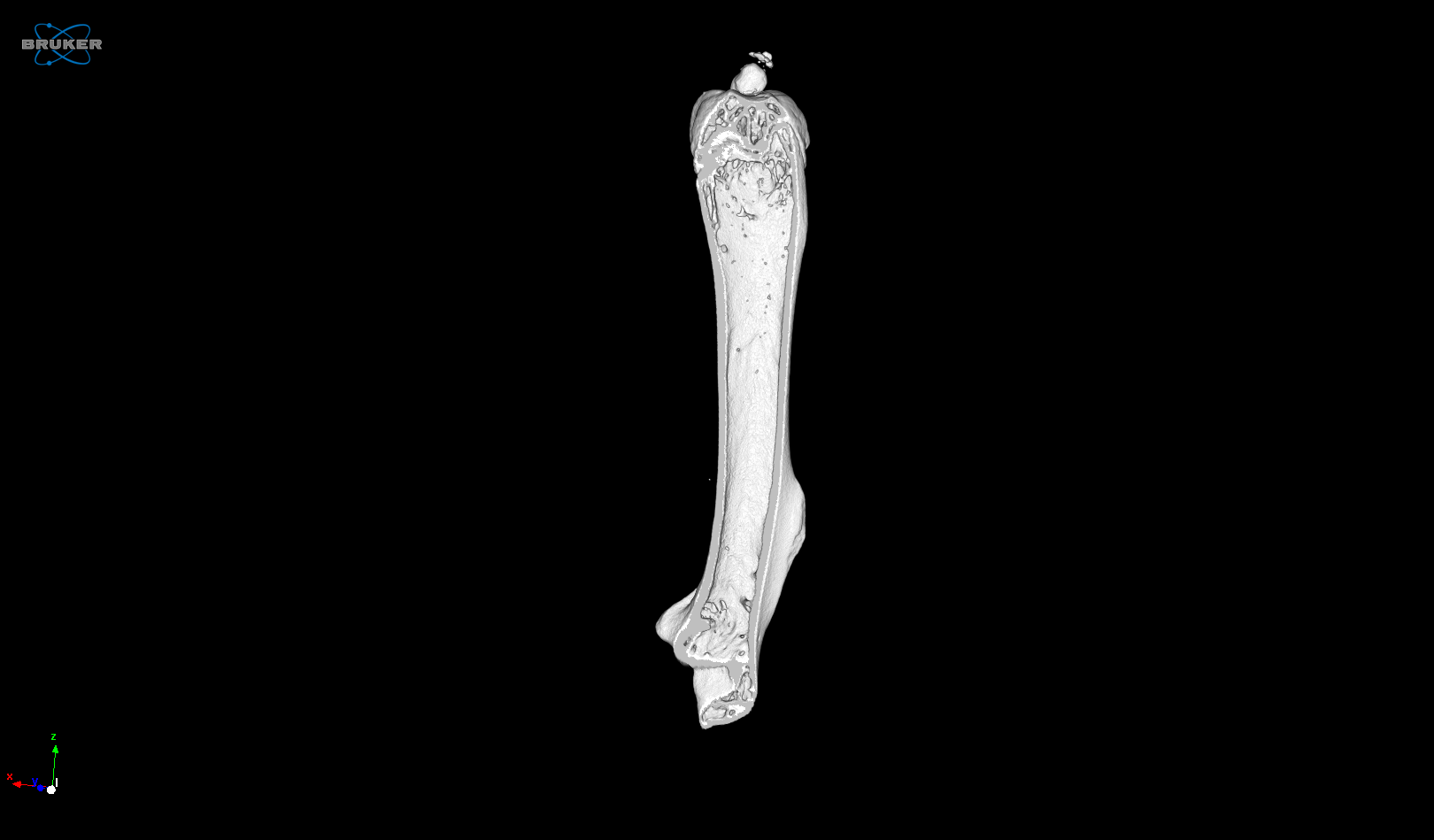

Supplement: S1 File — (ZIP) [file pone.0336703.s001.zip › Supporting Information Captions/S3_Data_MicroCT_quantification/Picture/9-week model/9week-1-1.bmp]

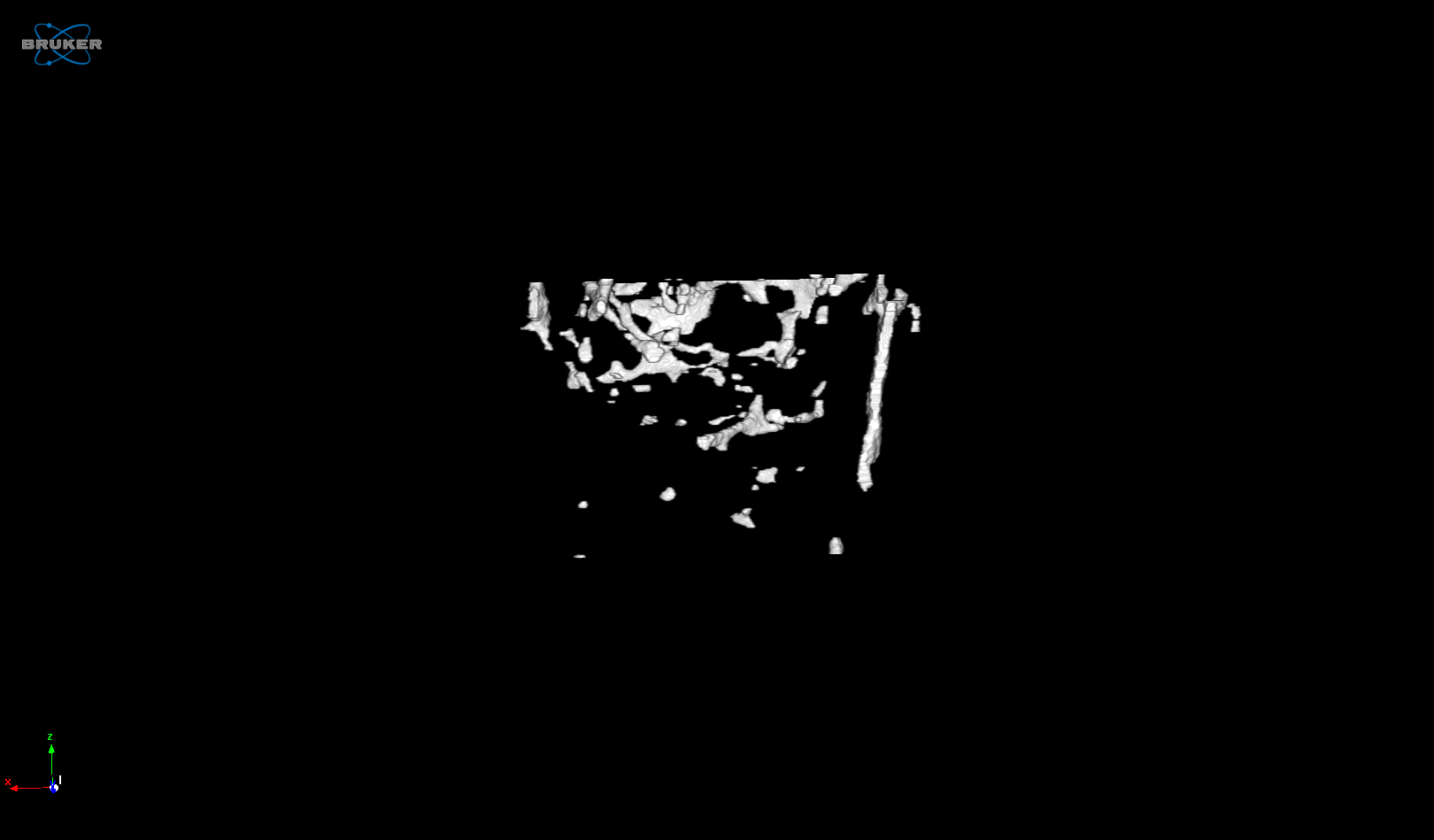

Supplement: S1 File — (ZIP) [file pone.0336703.s001.zip › Supporting Information Captions/S3_Data_MicroCT_quantification/Picture/9-week model/9week-1-2.bmp]

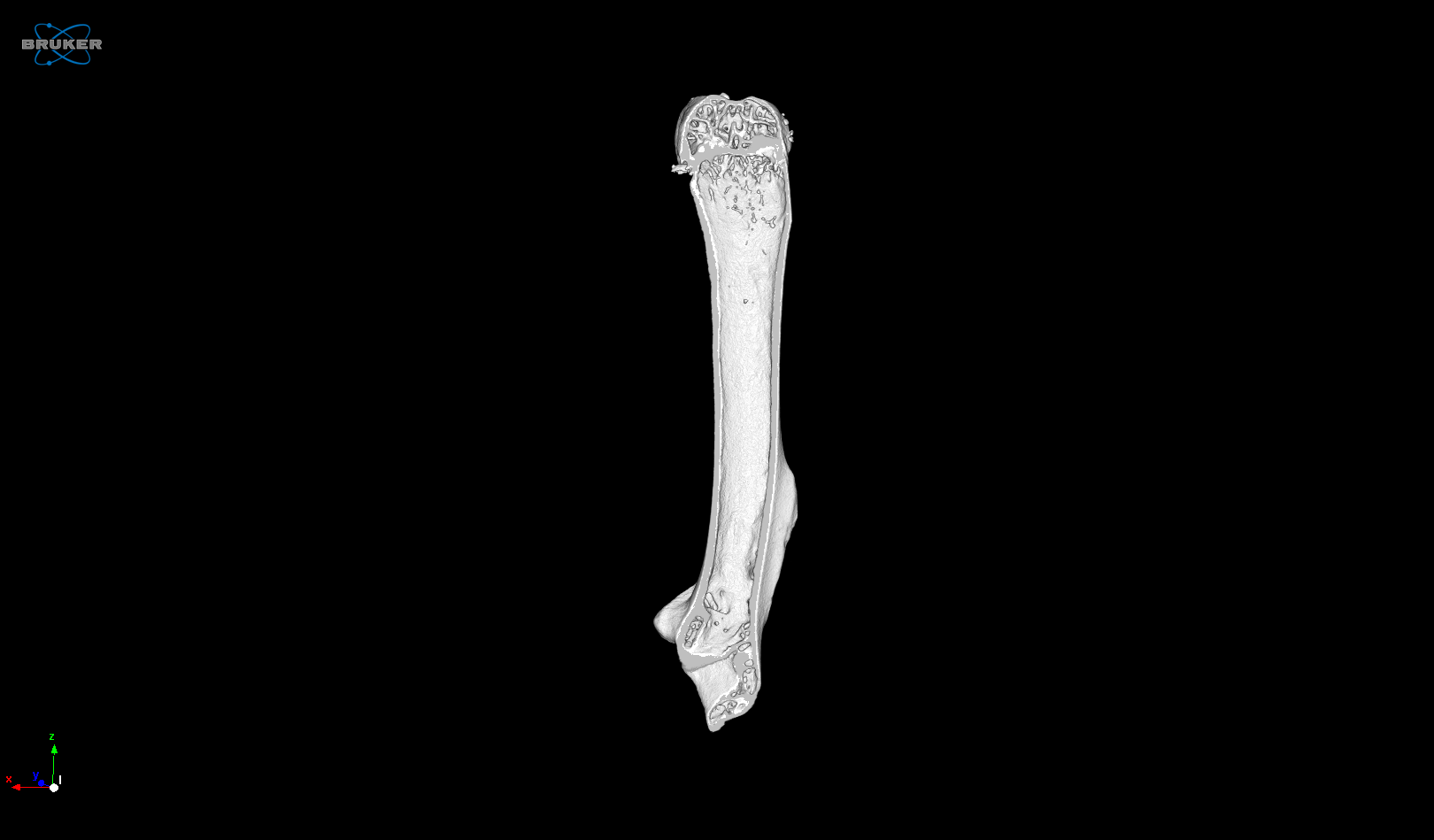

Supplement: S1 File — (ZIP) [file pone.0336703.s001.zip › Supporting Information Captions/S3_Data_MicroCT_quantification/Picture/9-week model/9week-2-1.bmp]

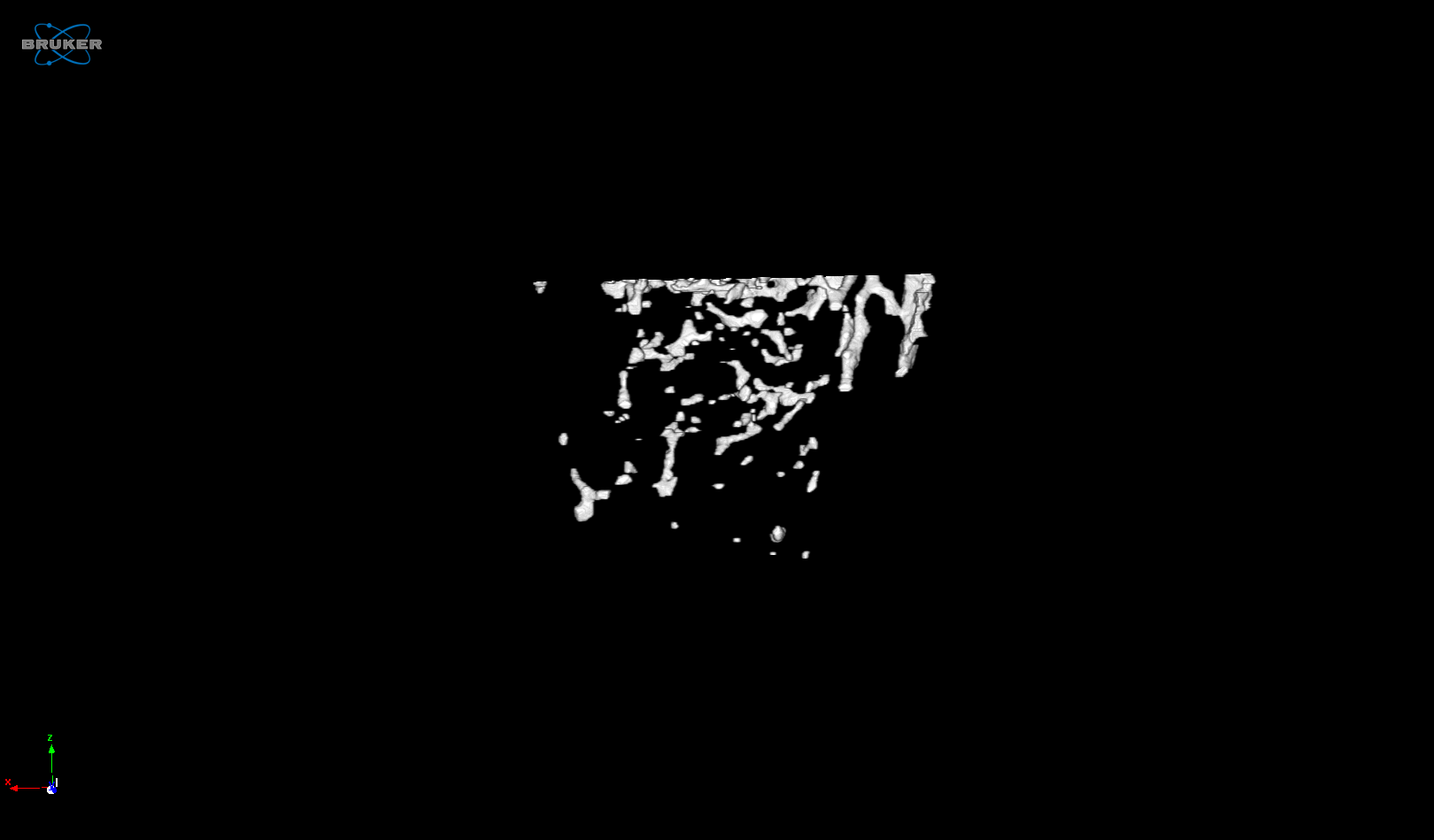

Supplement: S1 File — (ZIP) [file pone.0336703.s001.zip › Supporting Information Captions/S3_Data_MicroCT_quantification/Picture/9-week model/9week-2-2.bmp]

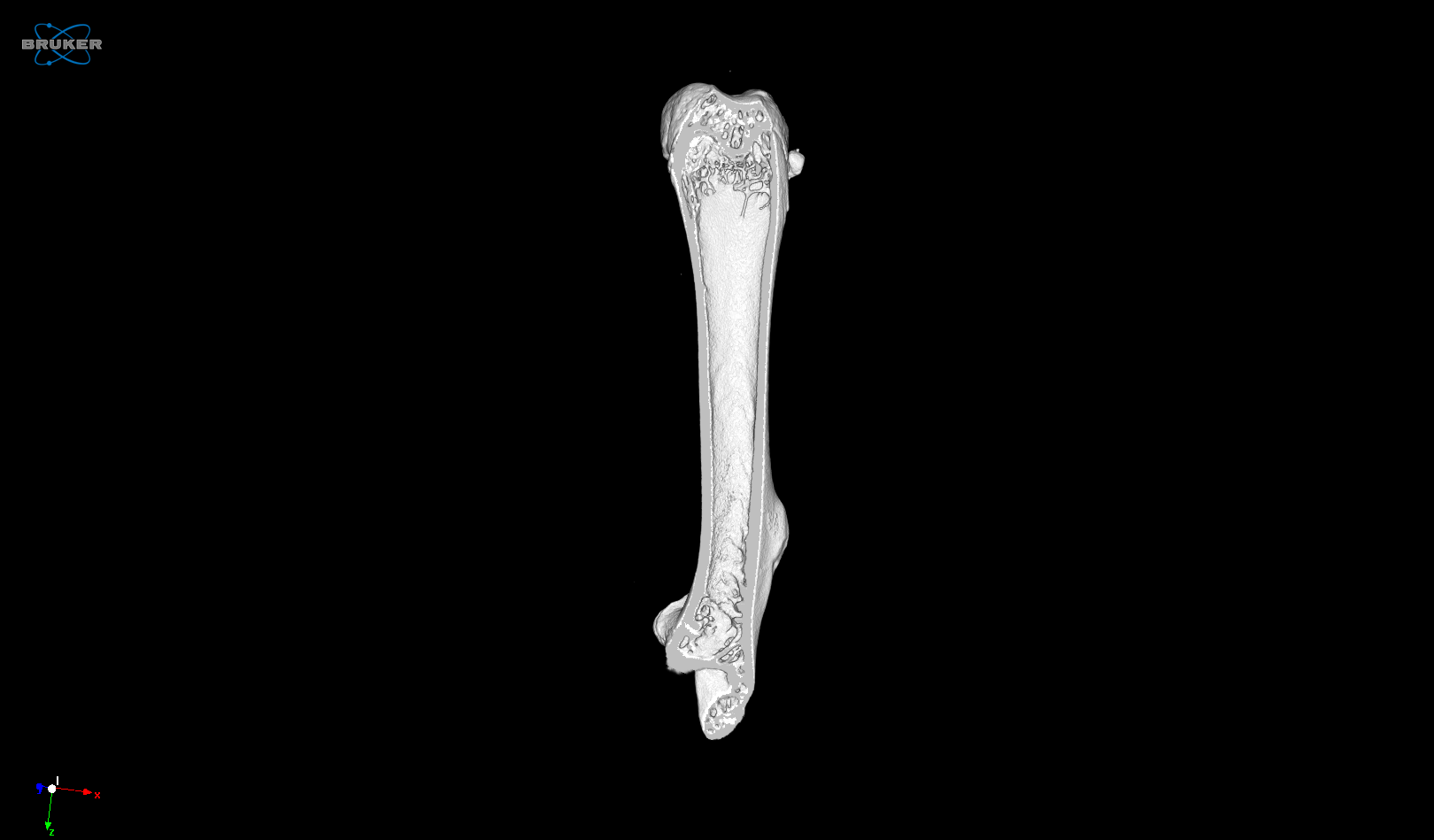

Supplement: S1 File — (ZIP) [file pone.0336703.s001.zip › Supporting Information Captions/S3_Data_MicroCT_quantification/Picture/9-week model/9week-3-1.bmp]

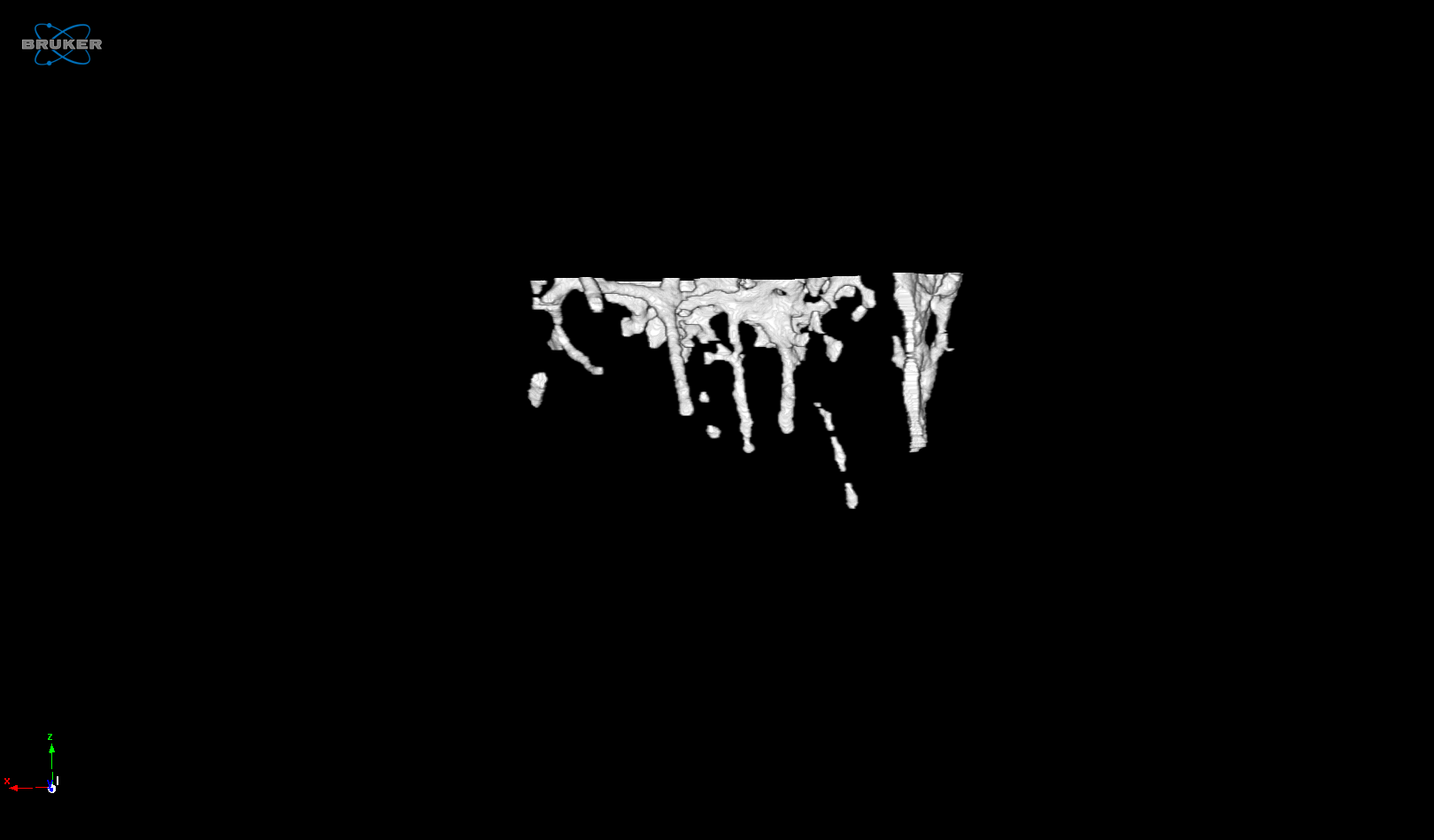

Supplement: S1 File — (ZIP) [file pone.0336703.s001.zip › Supporting Information Captions/S3_Data_MicroCT_quantification/Picture/9-week model/9week-3-2.bmp]

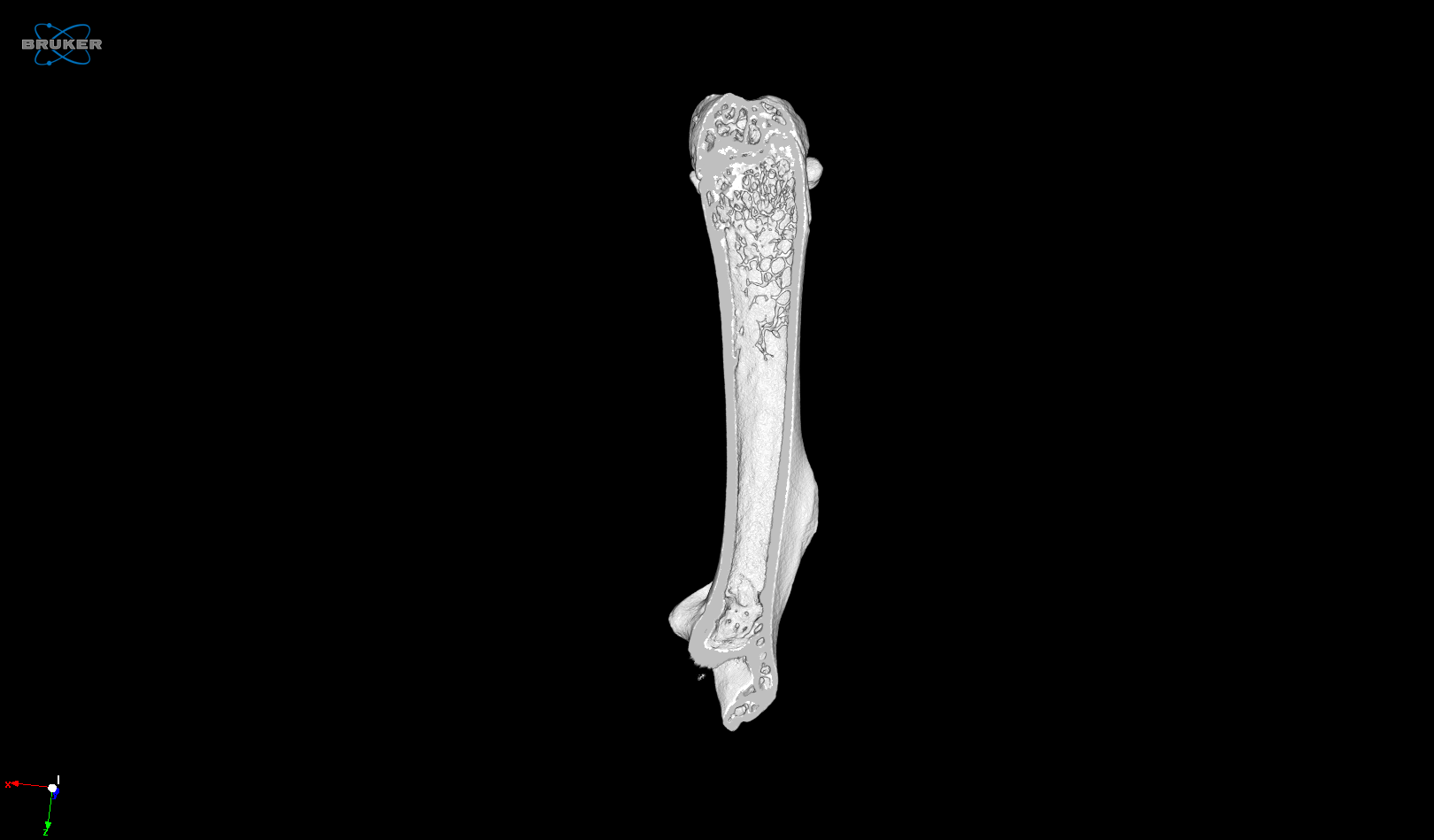

Supplement: S1 File — (ZIP) [file pone.0336703.s001.zip › Supporting Information Captions/S3_Data_MicroCT_quantification/Picture/sham/sham-1-1.bmp]

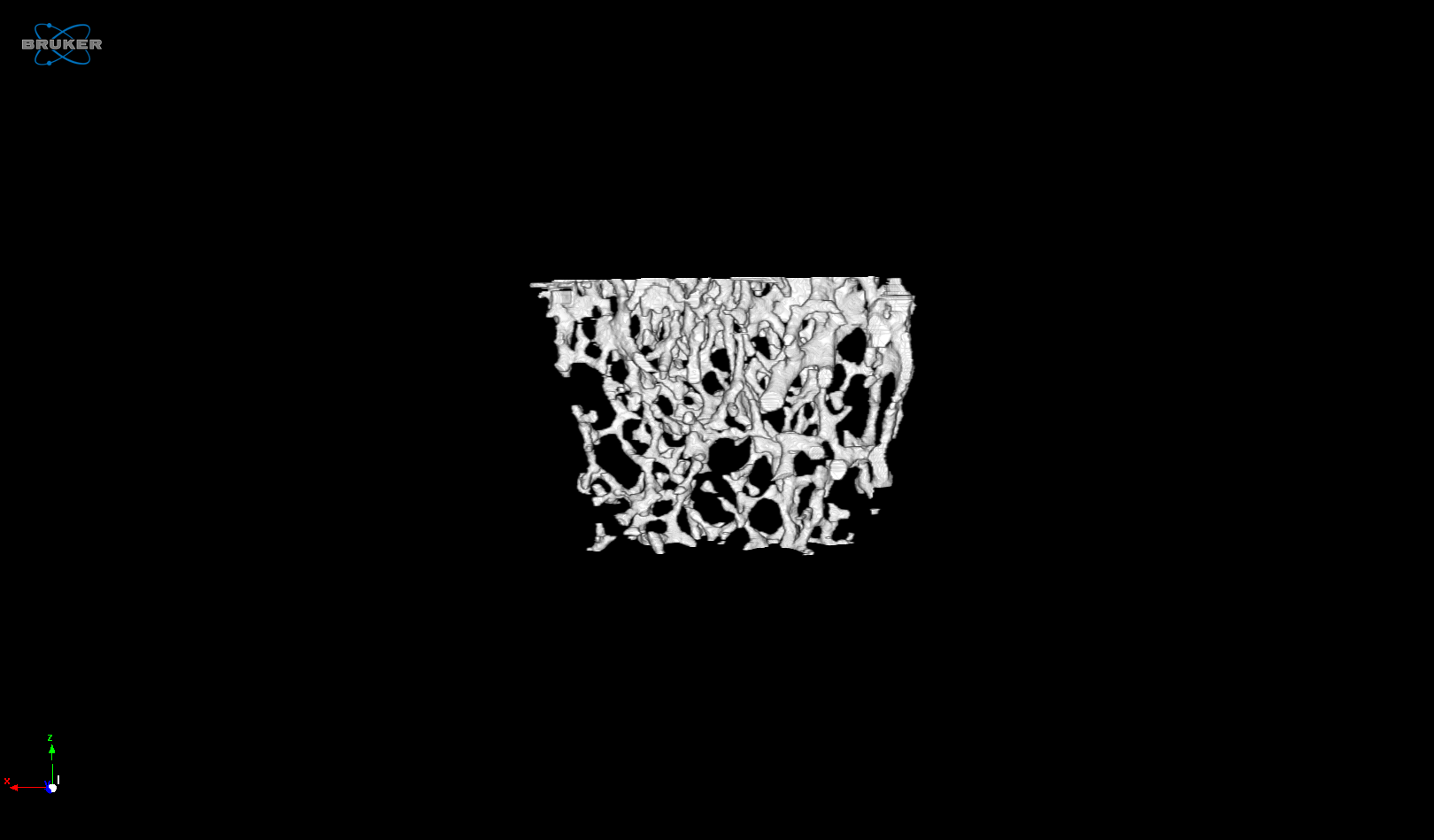

Supplement: S1 File — (ZIP) [file pone.0336703.s001.zip › Supporting Information Captions/S3_Data_MicroCT_quantification/Picture/sham/sham-1-2.bmp]

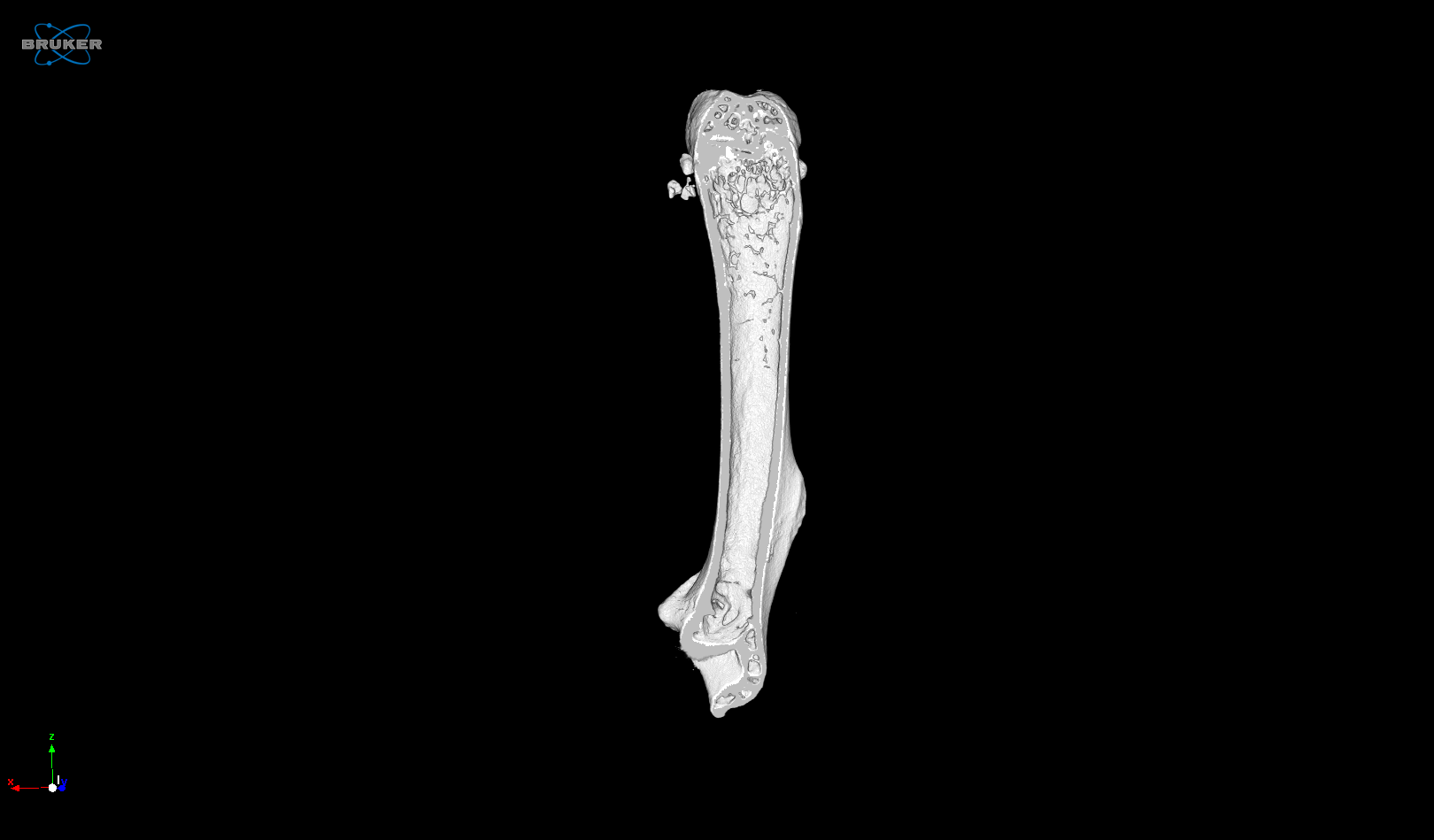

Supplement: S1 File — (ZIP) [file pone.0336703.s001.zip › Supporting Information Captions/S3_Data_MicroCT_quantification/Picture/sham/sham-2-1.bmp]

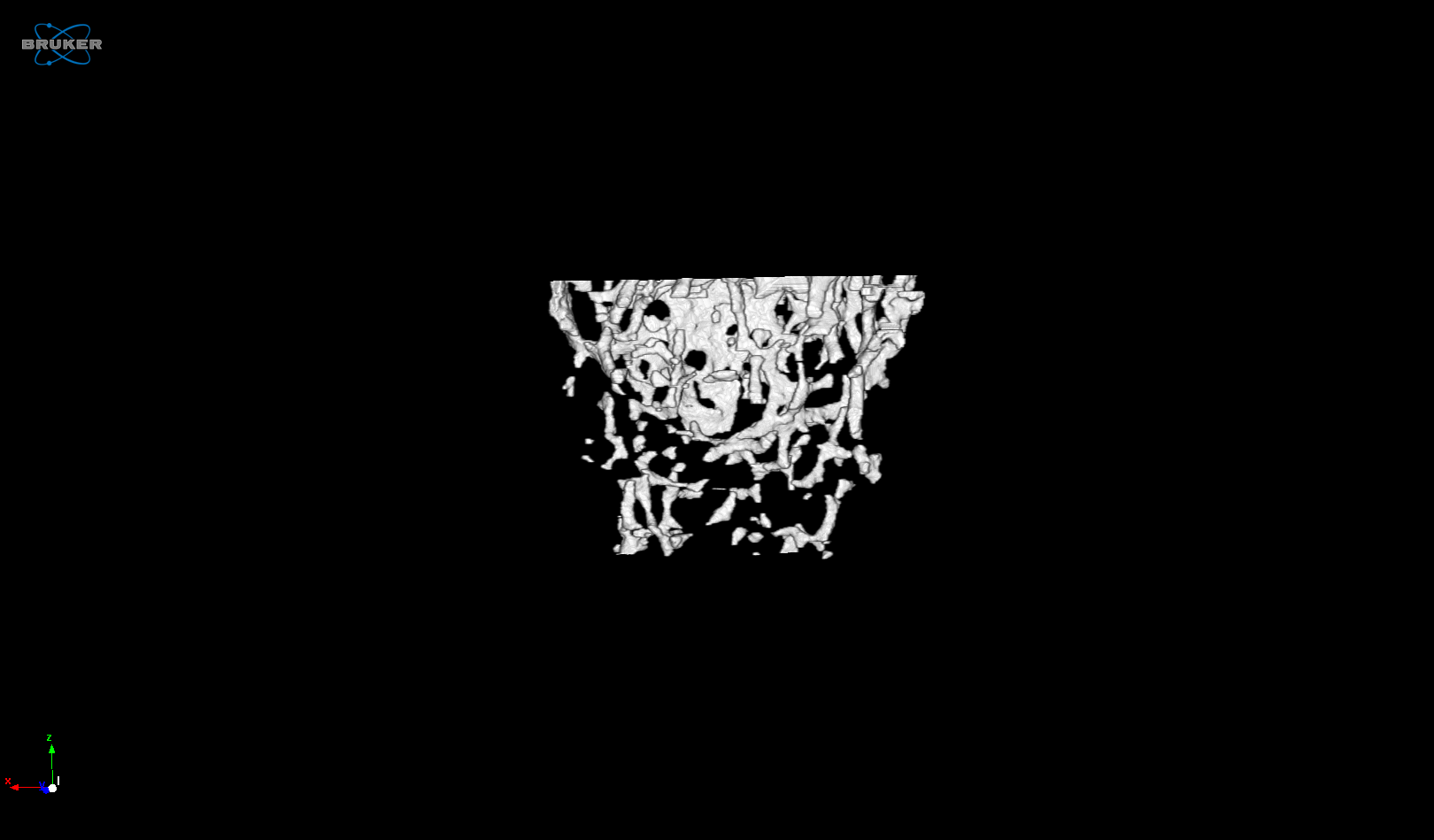

Supplement: S1 File — (ZIP) [file pone.0336703.s001.zip › Supporting Information Captions/S3_Data_MicroCT_quantification/Picture/sham/sham-2-2.bmp]

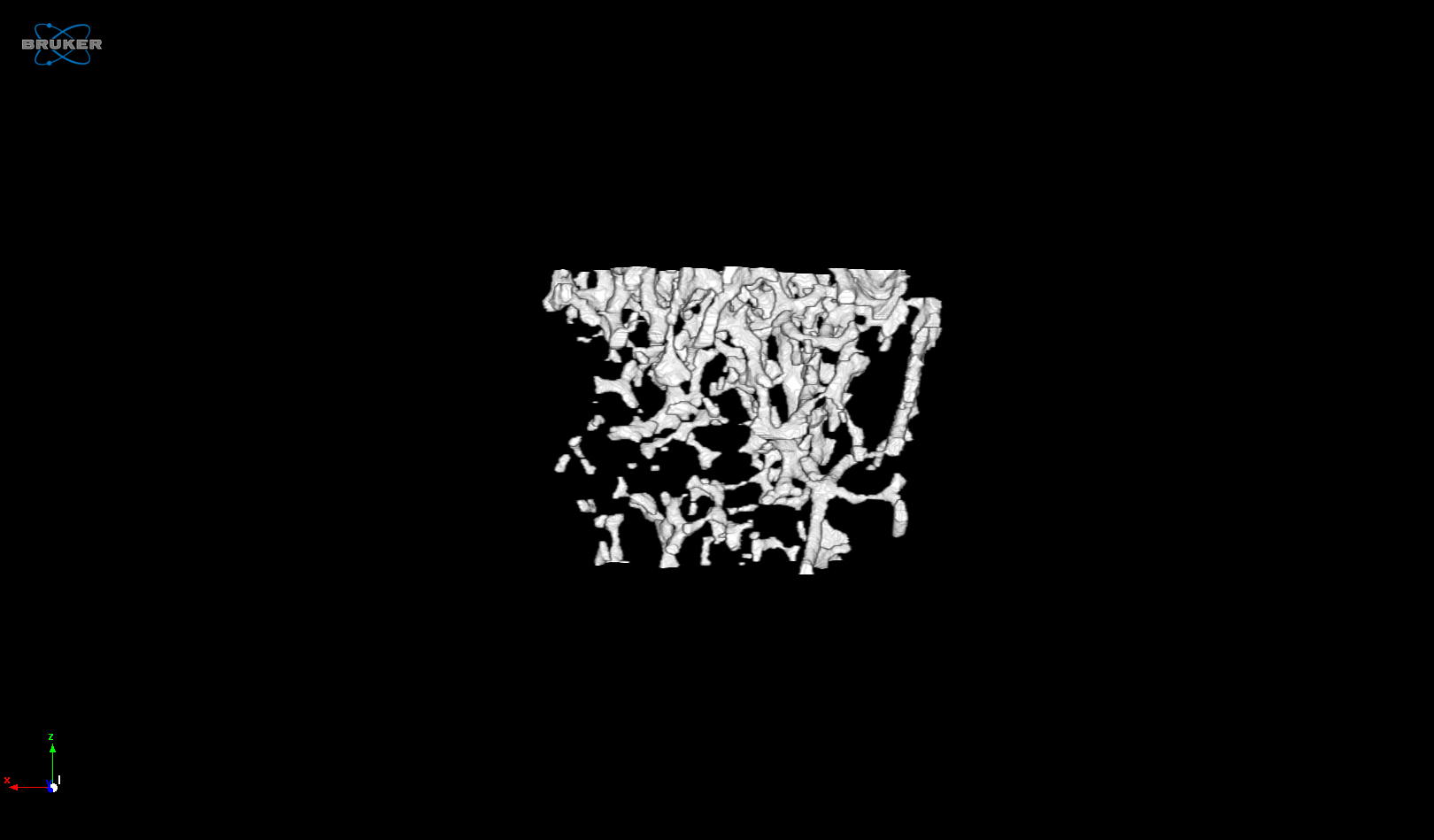

Supplement: S1 File — (ZIP) [file pone.0336703.s001.zip › Supporting Information Captions/S3_Data_MicroCT_quantification/Picture/sham/sham-3-1.bmp]

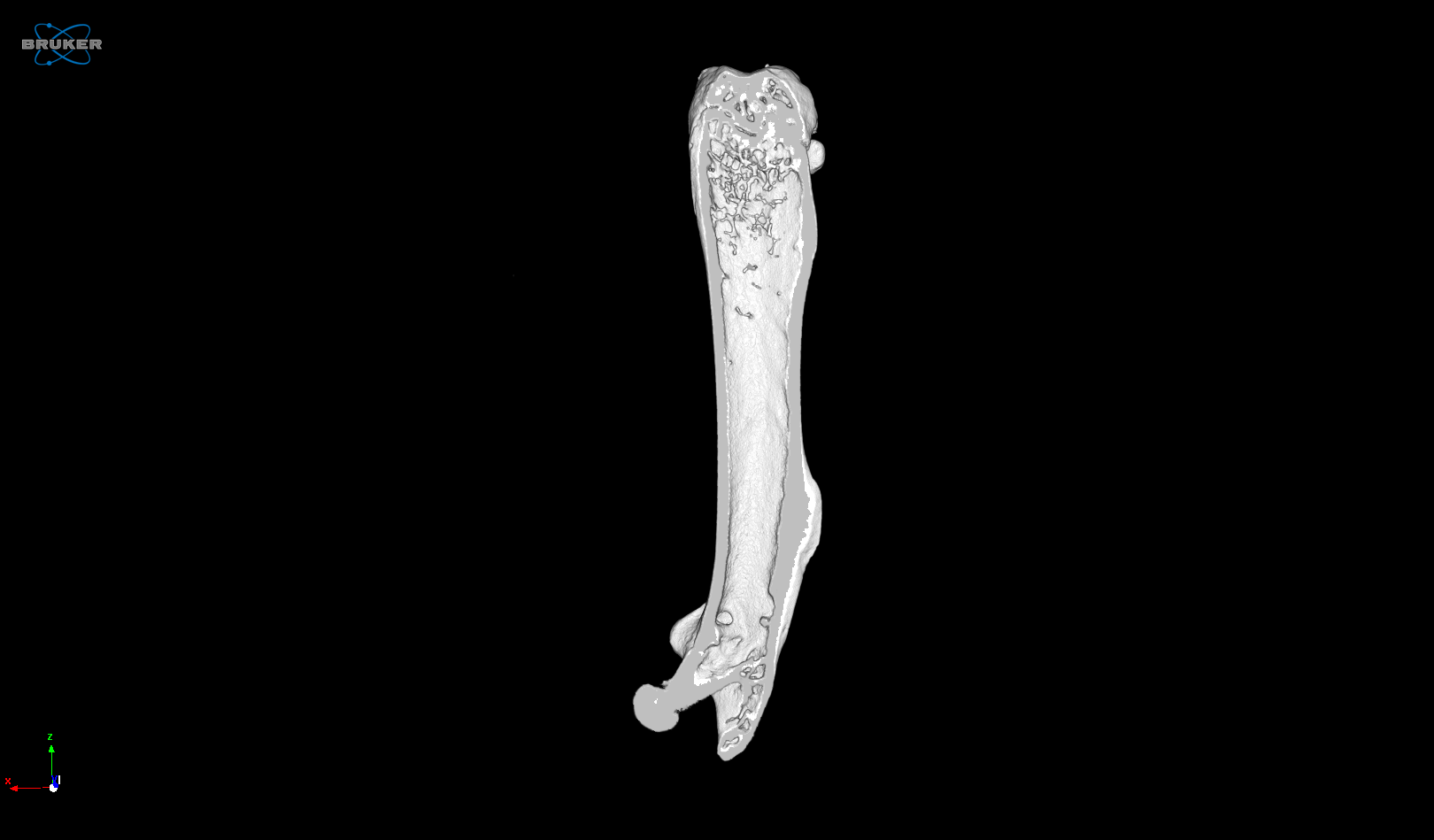

Supplement: S1 File — (ZIP) [file pone.0336703.s001.zip › Supporting Information Captions/S3_Data_MicroCT_quantification/Picture/sham/sham-3-2.bmp]
